# A novel approach to characterize phenotypic variation in GSD IV: Reconceptualizing the clinical continuum

**DOI:** 10.3389/fgene.2022.992406

**Published:** 2022-09-13

**Authors:** Bridget T. Kiely, Rebecca L. Koch, Leticia Flores, Danielle Burner, Samantha Kaplan, Priya S. Kishnani

**Affiliations:** ^1^ Duke University Medical Center, Department of Pediatrics, Division of Medical Genetics, Durham, NC, United States; ^2^ Medical Center Library and Archives, Duke University School of Medicine, Durham, NC, United States

**Keywords:** glycogen storage disease type IV, GSD IV, glycogenosis IV, Andersen disease, glycogen branching enzyme, GBE1

## Abstract

**Purpose:** Glycogen storage disease type IV (GSD IV) has historically been divided into discrete hepatic (classic hepatic, non-progressive hepatic) and neuromuscular (perinatal-congenital neuromuscular, juvenile neuromuscular) subtypes. However, the extent to which this subtype-based classification system accurately captures the landscape of phenotypic variation among GSD IV patients has not been systematically assessed.

**Methods:** This study synthesized clinical data from all eligible cases of GSD IV in the published literature to evaluate whether this disorder is better conceptualized as discrete subtypes or a clinical continuum. A novel phenotypic scoring approach was applied to characterize the extent of hepatic, neuromuscular, and cardiac involvement in each eligible patient.

**Results:** 146 patients met all inclusion criteria. The majority (61%) of those with sufficient data to be scored exhibited phenotypes that were not fully consistent with any of the established subtypes. These included patients who exhibited combined hepatic-neuromuscular involvement; patients whose phenotypes were intermediate between the established hepatic or neuromuscular subtypes; and patients who presented with predominantly cardiac disease.

**Conclusion:** The application of this novel phenotypic scoring approach showed that–in contrast to the traditional subtype-based view–GSD IV may be better conceptualized as a multidimensional clinical continuum, whereby hepatic, neuromuscular, and cardiac involvement occur to varying degrees in different patients.

## 1 Introduction

Glycogen storage disease type IV (GSD IV) is an autosomal recessive disorder caused by deficiency of glycogen branching enzyme (GBE), a ubiquitously-expressed enzyme that catalyzes the formation of alpha 1,6 branch points during glycogen synthesis (Brown and Brown, 1966; Bruno et al., 2004; Magoulas and El-Hattab, 2019). Absent or diminished activity of this enzyme leads to the accumulation of linear, amylopectin-like glycogen molecules (polyglucosan bodies) that are relatively insoluble due to the absence of normal branching patterns. The accumulation of polyglucosan bodies is thought to cause damage to cells *via* a foreign body reaction or osmotic mechanism, although much remains unknown about the pathophysiology of this disorder (Howell, 1991; Magoulas and El-Hattab, 2019).

Clinically, GSD IV is characterized by a remarkable degree of phenotypic heterogeneity. Although the first known reports of this disorder in the 1950s and 1960s described patients with progressive, fatal hepatic disease (Andersen, 1956; Sidbury et al., 1962; Holleman et al., 1966; Fernandes and Huijing, 1968), the range of phenotypes known to be associated with this disorder was subsequently expanded to include patients with predominantly neuromuscular or cardiac manifestations (Servidei et al., 1987; Reusche et al., 1992; Herrick et al., 1994; Tang et al., 1994) and patients with milder hepatic disease (Greene et al., 1988; McConkie-Rosell et al., 1996). Adult polyglucosan body disease (APBD)—an adult-onset disorder characterized by urinary incontinence, neuropathy, gait abnormalities, and other neurological disturbances—was also found to be caused by deficient GBE activity in the 1990s (Lossos et al., 1991). Despite sharing the same underlying enzymatic defect, GSD IV and APBD have traditionally been considered to represent distinct disorders, although this view has recently been challenged (Mochel et al., 2012; Paradas et al., 2014).

In recognition of the phenotypic heterogeneity of this disorder, GSD IV has been divided into several discrete clinical subtypes, which have been described in both the research literature and clinical references related to this disorder (Akman et al., 2006; Iijima et al., 2018; Szymanska et al., 2018; Magoulas and El-Hattab, 2019). Under the established subtype-based classification system, patients are categorized into either hepatic or neuromuscular subtypes that are then further sub-divided based on clinical severity and the rate of disease progression. Hepatic subtypes include the 1) severe “classic hepatic” form, which is typically defined to include patients with progressive liver dysfunction leading to death or a need for liver transplant (LT) within the first 5 years of life (Magoulas et al., 2012; Magoulas and El-Hattab, 2019; Sreekantam et al., 2020); and 2) the milder, “non-progressive hepatic” subtype, which is less clearly defined and includes patients with stable hepatopathy that does not progress to severe liver failure requiring transplantation by age 5 years (McConkie-Rosell et al., 1996; Iijima et al., 2018; Ichimoto et al., 2020). Neuromuscular subtypes include the 1) “perinatal neuromuscular” and 2) “congenital neuromuscular” subtypes, which have been recognized as distinct from one another by some authors (Bruno et al., 2004; Akman et al., 2006; Magoulas and El-Hattab, 2019) and combined into a single group by others (Maruyama et al., 2004; Iijima et al., 2018). Both subtypes (hereafter referred to as “perinatal-congenital neuromuscular”) are characterized by the presence of profound skeletal muscle weakness that is evident at or before birth, leading to respiratory failure and death during early infancy. The 3) “childhood neuromuscular” or “juvenile neuromuscular” subtype (hereafter referred to as “juvenile neuromuscular”) is considered less severe and follows a protracted clinical course characterized by chronic, progressive myopathy and exercise intolerance during childhood (Reusche et al., 1992; Schröder et al., 1993). Cardiomyopathy has been reported to occur in association with all of the aforementioned subtypes (Reusche et al., 1992; Janecke et al., 2004; Willot et al., 2010; Aksu et al., 2012).

At least two key assumptions are implicit in the established subtype-based classification system. The first is that most patients with GSD IV exhibit either predominantly hepatic or predominantly neuromuscular manifestations. The second is that phenotypic variation is clustered into discrete, mutually exclusive subgroups within the hepatic and neuromuscular domains, rather than varying along a continuum. The validity of these assumptions has been challenged by reports of patients with GSD IV whose phenotypes did not fully correspond with any of the existing subtypes (Burrow et al., 2006; Malfatti et al., 2016; Szymanska et al., 2018; Ndugga-Kabuye et al., 2019), prompting some authors to question whether GSD IV is better conceptualized as a clinical continuum rather than as discrete subtypes (Burrow et al., 2006; Derks et al., 2021). However, this claim has not been evaluated systematically, since clinical studies of this disorder have, to date, been limited primarily to individual case reports and small case series. Therefore, this study conducted an analysis of all published reports of patients with a confirmed diagnosis of GSD IV presenting during childhood or early adulthood. A novel approach to phenotypic characterization was applied to these cases in order to evaluate the validity of the established subtype-based classification system and to comprehensively describe the multisystem involvement in GSD IV.

## 2 Materials and methods

### 2.1 Literature search and data extraction

**FIGURE 1:**
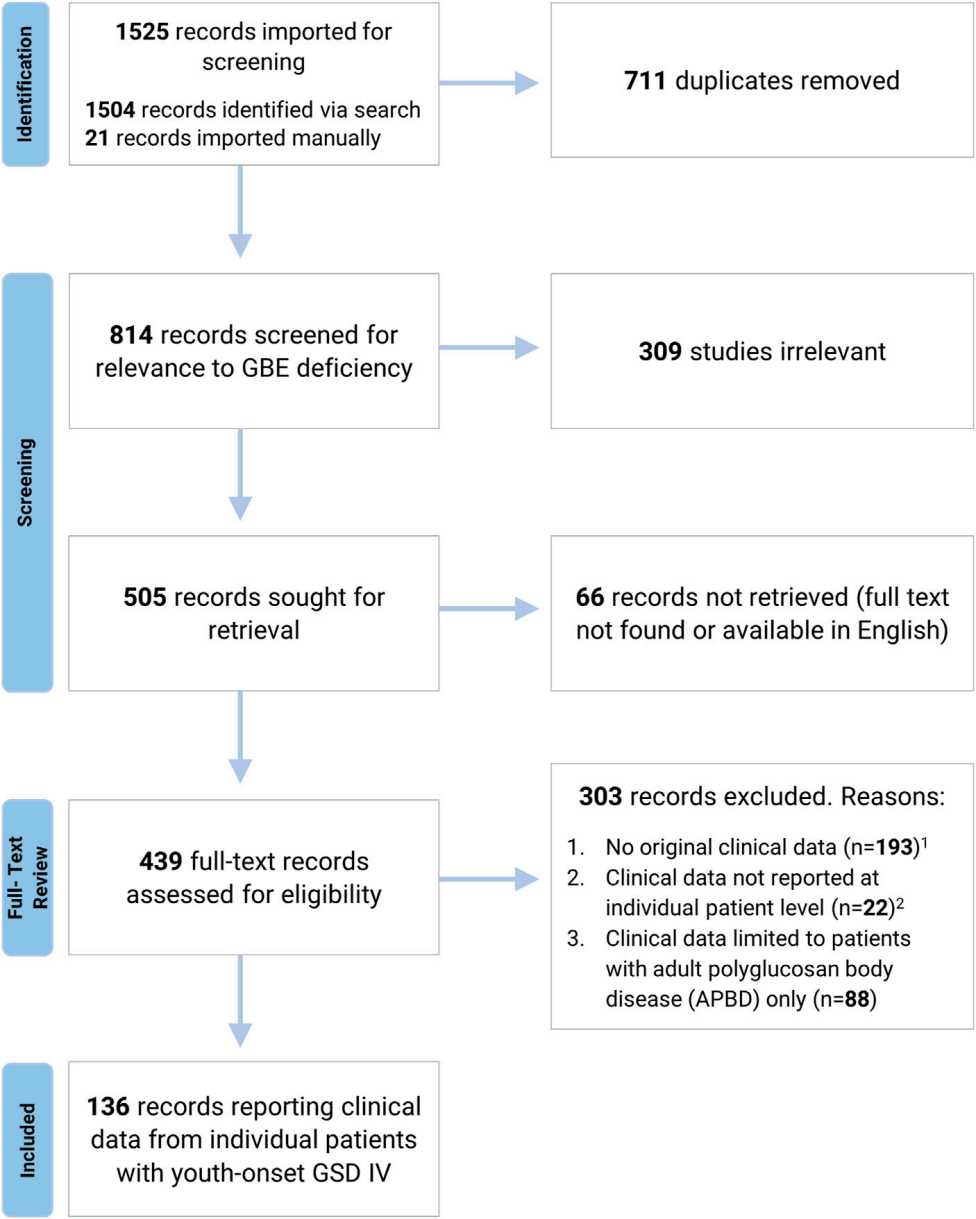
Systematic Review Process. A systematic search of MEDLINE *via* Ovid, Embase *via* Elsevier, and Scopus *via* Elsevier was undertaken to identify all published cases of GSD IV. Search strategy is detailed in Supplementary Appendix S1. ^1^ Pre-clinical studies, review articles, and papers reporting biochemical or genetic data without associated clinical findings were excluded due to a lack of original clinical data. ^2^ Records were excluded if clinical data were reported in aggregate or if patients were not individually identified *via* the use of unique labels and/or table entries (for records reporting data from more than one GSD IV patient).

A systematic search of the literature was undertaken to identify all published cases of GSD IV. Using a strategy designed by a Duke University School of Medicine medical librarian, the databases MEDLINE *via* Ovid, Embase *via* Elsevier, and Scopus *via* Elsevier were searched for relevant publications, as detailed in Figure 1 and Supplementary Appendix S1. References identified by this search were imported into Covidence Systematic Review Software (Veritas Health Innovation, Melbourne, Australia) for further review. After duplicate references and papers not relevant to GSD IV were excluded, the remaining papers were evaluated by two independent reviewers to assess their eligibility for inclusion. Conflicts were resolved by discussion with input from a third reviewer. Papers were excluded if the full text could not be located in English, if no clinical data were reported, or if clinical data were reported in aggregate rather than at the individual patient level. Papers exclusively reporting data on patients with the adult-onset form of GSD IV (APBD) were also excluded, as the aim of the current study was to characterize phenotypic variation among patients with onset of GSD IV symptoms during the first 24 years of life (hereafter referred to as “youth-onset GSD IV”). All patients identified *via* this strategy were assigned an identification number for the purposes of the current review (Supplementary Tables S1, S2, S3). Each identification number had a preceding “L” for “literature.” Patients who were reported more than once in the published literature were assigned a single identification number, and data were extracted from all relevant publications to capture their comprehensive medical history. The final analyses were limited to patients for whom the diagnosis of GSD IV was established *via* identification of biallelic *GBE1* mutations and/or enzymatic confirmation of reduced GBE activity, as reported by the original authors. Patients diagnosed based solely on histological findings (i.e., without genetic or enzymatic confirmation) were included only if they had a sibling with an enzymatically- or genetically-confirmed diagnosis. These criteria were applied so as to avoid the inclusion of patients with mutations in other genes—such as *GYG1*, *RBCK1*, *PFKM*, *PRKAG2*, *LAMP2*, *EPM2A*, *NHLRC1*, and others—that may produce clinical and/or histological findings similar to those observed in GSD IV (Akman et al., 2020). For each patient meeting the inclusion criteria, all publications describing the patient were reviewed and relevant data were extracted into a Qualtrics (Qualtrics, Provo, UT) form, which was then exported directly into an Excel (Microsoft^®^ Excel^®^ 2019 version 1808) spreadsheet.

### 2.2 Assessment of multisystem involvement

**FIGURE 2:**
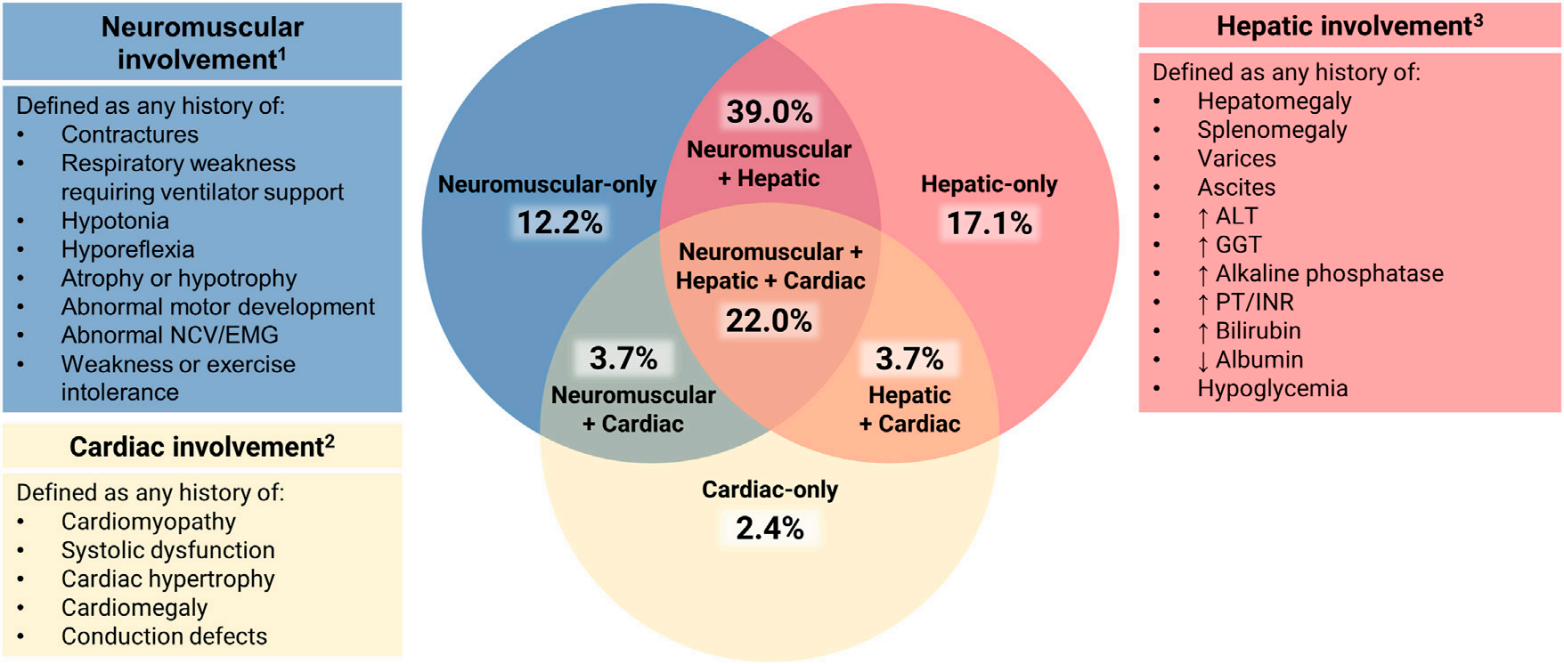
Assessment of Multisystem Involvement in GSD IV. All patients with available data were characterized based on their reported history of disease expression (present or absent) in three systems: hepatic (H), neuromuscular (N), and cardiac (C). Those with sufficient data to assess all three systems (*n* = 82) were represented on a Venn diagram in the following categories: neuromuscular-only (N present, H and C absent); hepatic-only (H present, N and C absent); cardiac-only (C present, H and N absent); neuromuscular + hepatic (H and N present, C absent); hepatic + cardiac (H and C present, N absent); neuromuscular + cardiac (N and C present, H absent); or hepatic + neuromuscular + cardiac (all three present). ^1^ Neurological abnormalities that could not be definitively attributed to GSD IV (e.g., childhood epilepsy, isolated language delay) were not used for characterization purposes. ^2^ Structural cardiac defects other than cardiomyopathy (e.g., ventricular septal defects, double aortic arch) were not used for characterization purposes due to their unclear association with GSD IV. ^3^ The following findings, if present, were not used for hepatic characterization: 1) hypoglycemia and/or hyperbilirubinemia limited to the immediate newborn period; 2) ascites that occurred exclusively in the setting of heart failure.

For all patients with available data, disease expression was assessed across three systems: hepatic, neuromuscular, and cardiac. Patients who died *in utero* were not scored. Clinical involvement of each of these systems was scored as present or absent based on the following criteria (Figure 2).

#### 2.2.1 Hepatic involvement

Defined as present if the patient had any current or prior history of hepatomegaly, splenomegaly, varices, ascites, and/or laboratory abnormalities [elevated alanine aminotransferase (ALT), gamma-glutamyl transferase (GGT), alkaline phosphatase, prothrombin time (PT)/international normalized ratio (INR), bilirubin, low albumin, or fasting hypoglycemia].

Defined as absent if the authors stated that there was no evidence of hepatic involvement or if they stated that the results of routine laboratory tests and/or abdominal imaging were normal at all reported visits.

#### 2.2.2 Neuromuscular involvement

Defined as present if the patient had any current or prior history of contractures, respiratory weakness requiring ventilator support, hypotonia, hyporeflexia, atrophy or hypotrophy, abnormal motor development, abnormal electromyography (EMG) or nerve conduction velocities (NCV), and/or weakness or exercise intolerance.

Defined as absent if the original authors stated that the patient’s development and/or neuromuscular examination were normal at all reported visits.

#### 2.2.3 Cardiac involvement

Defined as present if the patient had any current or prior history of cardiomyopathy, systolic dysfunction, cardiac hypertrophy, cardiomegaly, and/or conduction defects, as assessed by imaging, gross inspection on autopsy, or other testing such as electrocardiography (ECG).

Defined as absent if the original authors stated that the patient had no evidence of cardiac involvement or if they reported that the results of standard cardiac evaluations (e.g., ECG, echocardiogram) were normal at all reported visits.

### 2.3 Phenotypic scoring

In addition to being scored as present or absent, hepatic and neuromuscular disease manifestations were further characterized in order to evaluate the extent to which each patient’s overall phenotype was consistent with one of the established subtypes. To facilitate systematic phenotypic characterization, the definitions of the established subtypes were operationalized into four-point hepatic (H0-H3) and neuromuscular (N0-N3) phenotypic scoring scales, which were used to assign all patients with available data a two-part score consisting of hepatic and neuromuscular components.

**FIGURE 3:**
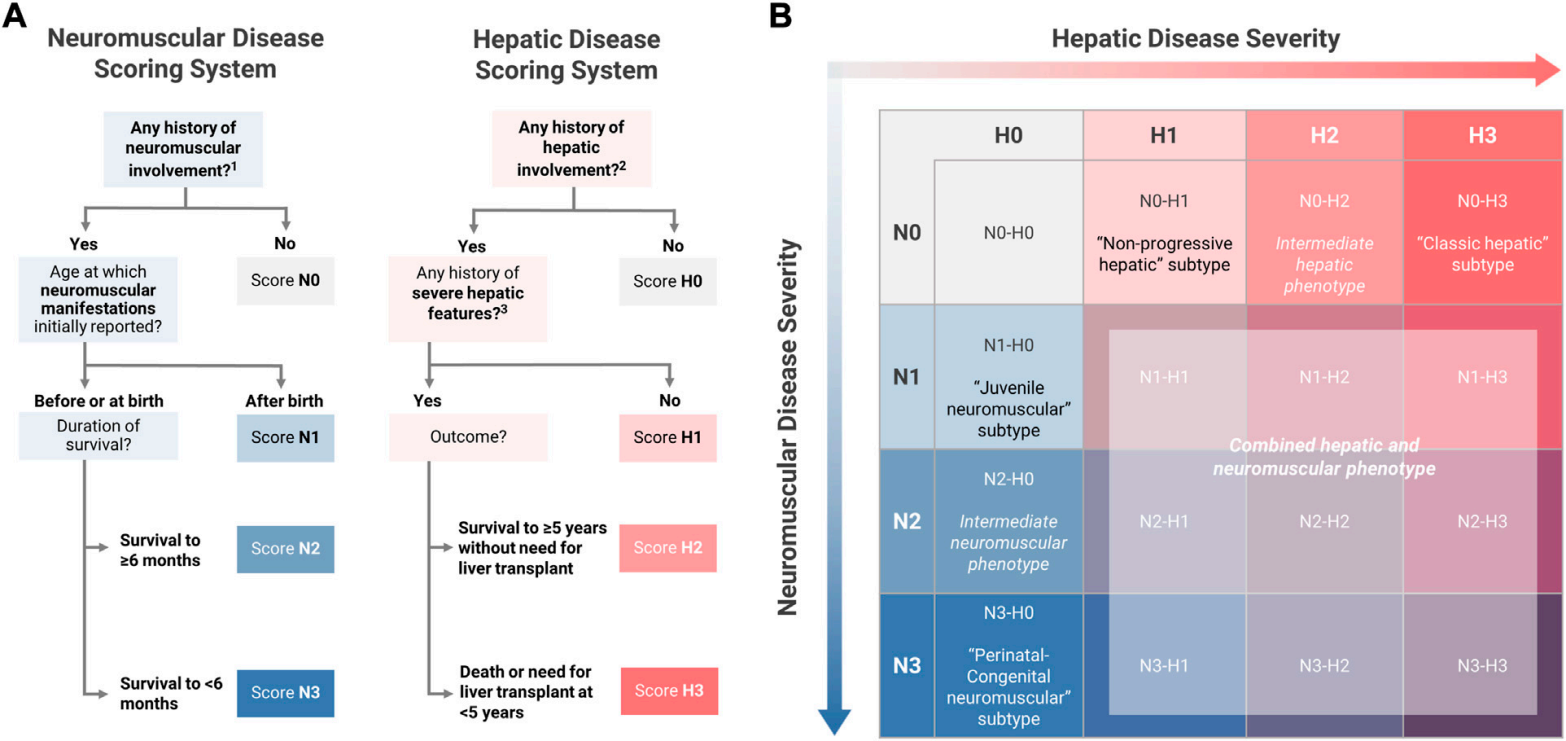
Novel Approach to Phenotypic Characterization of GSD IV. **(A)** The definitions of the established subtypes for youth-onset GSD IV were used as the basis for defining four-point hepatic (H0-H3) and neuromuscular (N0-N3) scoring scales. All patients were assigned a two-part score with hepatic and neuromuscular components based on the scoring algorithms shown here. **(B)** Two-part scoring combinations were represented on a 4 × 4 matrix with hepatic scores on the horizontal axis and neuromuscular scores on the vertical axis. Under this proposed model, patients with the following scoring combinations were considered to be phenotypically consistent with one of the established subtypes: N1-H0 (“juvenile neuromuscular” subtype); N3-H0 (“perinatal-congenital neuromuscular” subtype); N0-H1 (“non-progressive hepatic” subtype); N0-H3 (“classic hepatic” subtype). All other scoring combinations describe phenotypes that are not fully consistent with any of the established subtypes, including intermediate phenotypes (N2-H0 and N0-H2); mixed hepatic-neuromuscular phenotypes (N>0-H>0); or cardiac-only phenotypes (N0-H0). ^1^ See Figure 2 for the definition of “any history of neuromuscular involvement.” ^2^ See Figure 2 for the definition of “any history of hepatic involvement.” ^3^ Definition of severe hepatic features: any history of liver failure or synthetic dysfunction; jaundice or hyperbilirubinemia; ascites; varices or gastrointestinal bleed; or splenomegaly with thrombocytopenia.

Within the neuromuscular domain, scoring criteria were defined such that the maximum score of N3 would be consistent with the perinatal-congenital neuromuscular subtype, while N1 would be consistent with the juvenile neuromuscular subtype. The criteria for the score of N2 were defined so as to include patients with phenotypes that were intermediate between the established neuromuscular subtypes. Since at least two major features—1) the timing of symptom onset, and 2) the duration of survival—distinguish the perinatal-congenital neuromuscular subtype from the juvenile neuromuscular subtype (Magoulas and El-Hattab, 2019), these two features were used as the basis for defining the scoring criteria, as detailed below (Figure 3):

**N3** (*perinatal-congenital neuromuscular*): Onset of neuromuscular manifestations **at or before birth**, leading to death at **<6 months of age**.
**N2** (*intermediate neuromuscular*): Onset of neuromuscular manifestations **at or before birth**, with survival to **≥6 months of age**.
**N1** (*juvenile neuromuscular*): Onset of neuromuscular manifestations **after** birth.

Within the hepatic domain, a parallel approach was used to establish the criteria for scores of H3 (consistent with the classic hepatic subtype) and H1 (consistent with the non-progressive hepatic subtype). The criteria for the score of H2 were defined so as to include patients with phenotypes that were intermediate between the established hepatic subtypes. Two key distinguishing features of the classic hepatic subtype—1) the severity of hepatic disease, and 2) requirement for LT within the first 5 years of life—were used as the basis for defining the hepatic scoring criteria (Magoulas and El-Hattab, 2019). Hepatic disease was defined as “severe” if the patient had any reported history of liver failure or synthetic dysfunction; jaundice or hyperbilirubinemia; ascites; varices or gastrointestinal bleed; or splenomegaly with thrombocytopenia (<150,000/µl; consistent with “definite” clinically evident portal hypertension) (Bass et al., 2019). Based on these definitions, patients were scored as follows (Figure 3):

**H3** (*classic hepatic*): Hepatic involvement **with severe features** leading to **LT or death at <5 years** of age.
**H2** (*intermediate hepatic*): Hepatic involvement **with severe features**, surviving to **≥5 years of age *without* LT**.
**H1** (*non-progressive hepatic*): Hepatic involvement **without severe features**

Under this proposed model, the established subtypes may be thought of as corresponding to scoring combinations of N3-H0 (perinatal-congenital neuromuscular subtype); N1-H0 (juvenile neuromuscular); N0-H3 (classic hepatic); and N0-H1 (non-progressive hepatic). All other possible scoring combinations correspond to phenotypes that are not fully described by the established subtype-based classification system, due to the presence of both hepatic and neuromuscular involvement (N>0 and H>0) and/or phenotypic features that are intermediate in severity between the established subtypes (N2 and/or H2). By applying this scoring system to the patients in the current study, it was possible to determine the frequency with which patients with youth-onset GSD IV—as reported in the published literature—exhibit phenotypes that are not fully consistent with the established subtypes.

## 3 Results

### 3.1 Systematic search

The systematic search identified 136 papers reporting individual-level clinical data on 179 unique patients with youth-onset GSD IV (Figure 1). Of these 179 patients, 146 met the full criteria for inclusion, with a genetically- and/or enzymatically-confirmed diagnosis (*n* = 139) or a histologically-confirmed diagnosis and an affected sibling whose diagnosis was confirmed *via* genetic testing or measurement of GBE activity (*n* = 7).

### 3.2 Clinical course and survival

As of the last reported follow-up, 43.9% of patients were living (median age at last report: 6 years, range: 11 months to 31 years) while 56.1% were deceased. Analysis of the timing and reported causes of death identified several clusters. 24.3% (*n* = 19) of all deaths occurred prenatally. Whereas deaths between 0 and 4 months of age (*n* = 35; 44.9% of all deaths) typically occurred in patients with profound neuromuscular weakness associated with ventilator dependence or cardiopulmonary failure, most of the patients that died between 6 months and 3 years of age (*n* = 18; 23.1% of all deaths) had a history of severe hepatic disease. Common causes of death in the latter group included liver failure or hemorrhage (L10, L24, L121, L114, and L139), complications associated with liver transplant (L29 and L30), progression of extrahepatic disease such as cardiomyopathy (L38 and L103), and other causes such as infection (L8 and L12). Only 7.7% of all deaths (*n* = 6) occurred after 4 years of age. These included three patients who died of infection; two patients in whom progressive cardiomyopathy contributed to death at 8 years (L22) and 20 years (L41) of age; and one patient who died of liver failure in the setting of hepatocellular carcinoma at 13 years of age (L21).

### 3.3 Disease expression by system

Clinical evidence of neuromuscular involvement, as defined previously, was reported to be present in 80.0% of the 110 patients with available data. Hepatic involvement was present in 81.8% of the 110 patients with available data. The frequency with which specific hepatic and neuromuscular disease manifestations were reported in this sample is shown in Supplementary Table S4. Cardiac involvement, as defined previously, was reported to be present in 37.9% of all patients with available data. There was substantial variation in the age at which cardiac abnormalities were initially detected, ranging from infancy to the third decade of life. In addition to cardiomyopathy, arrhythmias and conduction abnormalities were reported in several patients, including multiform ventricular arrhythmia (L145); ST segment or T wave changes (L89, L108, and L145); AV block (L22); long QT interval (L47); and atrial fibrillation (L145).

The 82 patients for whom sufficient data were available to assess disease expression in all three systems (hepatic, neuromuscular, cardiac) were plotted on a Venn diagram (Figure 2). Of these patients, 31.7% (*n* = 26) were reported to exhibit clinical involvement of just one out of three systems: hepatic-only (17.1%; *n* = 14), neuromuscular-only (12.2%; *n* = 10), or cardiac-only (2.4%; *n* = 2). An additional 46.3% (*n* = 38) exhibited involvement of two systems: hepatic and neuromuscular (39.0%; *n* = 32), hepatic and cardiac (3.7%; *n* = 3), or neuromuscular and cardiac (3.7%; *n* = 3). The remaining 22.0% (*n* = 18) were reported to exhibit hepatic, neuromuscular, and cardiac involvement.

### 3.4 Phenotypic scoring

**FIGURE 4:**
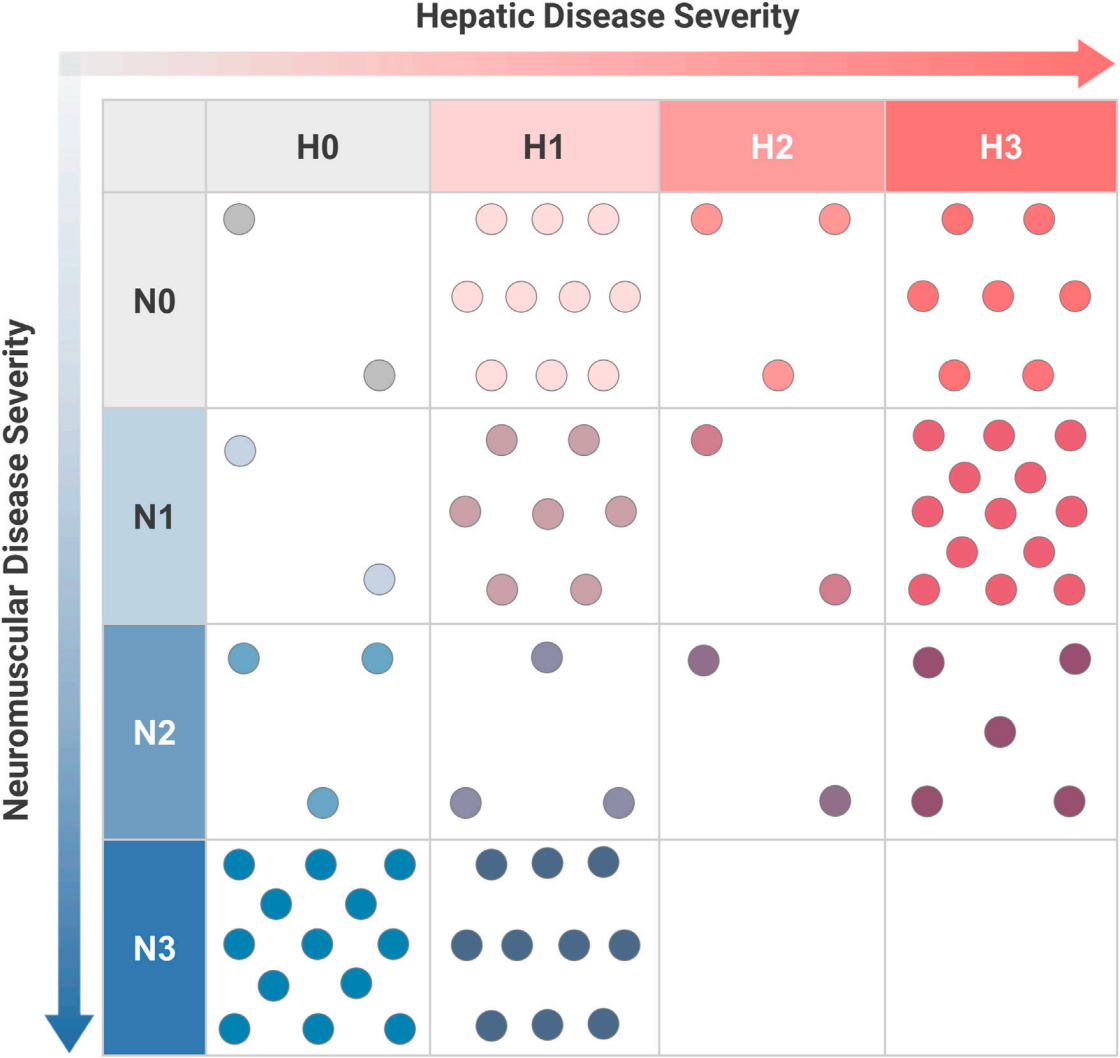
Distribution of Hepatic and Neuromuscular Phenotypes among Patients with GSD IV. For youth-onset GSD IV patients with sufficient data to calculate both neuromuscular (N) and hepatic (H) scores (*n* = 82), their combination of scores was plotted on a two-dimensional grid. Each dot corresponds to an individual patient. The established subtypes may be thought of as corresponding to scoring combinations of N3-H0 (“perinatal-congenital neuromuscular” subtype); N1-H0 (“juvenile neuromuscular” subtype); N0-H3 (“classic hepatic” subtype); and N0-H1 (“non-progressive hepatic” subtype). All other scoring combinations correspond to phenotypes that are not fully consistent with one of the established subtypes. Scoring criteria are detailed in Figure 3.

Sufficient data were available to assign a complete phenotypic score with hepatic and neuromuscular components to 82 patients. The distribution of hepatic-neuromuscular score combinations that were observed in this sample is shown in Figure 4. Analysis of the observed scoring combinations showed that 39.0% of these patients were phenotypically consistent with one of the established subtypes, as defined previously; these included score combinations of N0-H1 (*n* = 10, corresponding to the “non-progressive hepatic” subtype); N0-H3 (*n* = 7, “classic hepatic” subtype); N1-H0 (*n* = 2, “juvenile neuromuscular” subtype); N3-H0 (*n* = 13, “perinatal-congenital neuromuscular” subtype). The phenotypes of the remaining 61.0% of patients did not correspond fully with any of the established subtypes. These included patients who exhibited a combination of both hepatic and neuromuscular disease manifestations (N > 0 and H > 0; *n* = 42); patients who presented with phenotypes that were intermediate in severity between the established subtypes within the hepatic (N0-H2; *n* = 3) or neuromuscular (N2-H0; *n* = 3) domains; and patients who exclusively presented with cardiac disease without hepatic or neuromuscular involvement (N0-H0; *n* = 2).

## 4 Discussion

GSD IV is an ultra-rare autosomal recessive disorder caused by deficiency of GBE, a ubiquitously-expressed enzyme that is essential for the synthesis of normal glycogen branch points. Clinically, this disorder is characterized by a remarkable degree of phenotypic heterogeneity and has been described as consisting of distinct hepatic (classic or non-progressive) and neuromuscular (perinatal-congenital or juvenile) clinical subtypes (Magoulas and El-Hattab, 2019). However, the validity of this subtype-based classification system has not been evaluated empirically, and the published clinical literature on GSD IV has, to date, been primarily limited to individual case reports and small case series. This study synthesized clinical data from all eligible cases reported in the published literature to comprehensively capture the multisystem involvement in GSD IV. The use of a novel phenotypic scoring system in this study yielded a number of important insights that challenge the existence of discrete clinical subtypes and emphasize the importance of long-term, multidisciplinary clinical follow-up for all patients with GSD IV.

The first major conclusion of the current study is that many patients with GSD IV exhibit some degree of multisystem involvement. Whereas the established classification system divides patients into discrete hepatic or neuromuscular subtypes, the current study adopted a systematic approach to phenotypic characterization that assessed the extent of hepatic, neuromuscular, and cardiac involvement in each patient. This approach identified numerous patients with mixed phenotypes that are challenging to account for under the established subtype-based classification system. These included patients who presented at birth with evidence of neuromuscular weakness, followed by onset of progressive hepatic dysfunction leading to death or a requirement for transplant (L55, L143, and L12), consistent with features of both the congenital neuromuscular and classic hepatic subtypes. At the less severe end of the disease spectrum, several other patients presented with features that were consistent with elements of both the non-progressive hepatic and juvenile neuromuscular subtypes (L35, L36, L141) These findings suggest that hepatic and neuromuscular disease do not constitute mutually exclusive GSD IV subtypes, but rather, may be considered to represent distinct dimensions of phenotypic variation that are involved to different degrees in each patient. Compared to the traditional subtype-based view, this model better accounts for the wide range of phenotypes that were observed in this sample, including patients with exclusive hepatic or neuromuscular involvement; mixed hepatic and neuromuscular involvement; and neither hepatic nor neuromuscular involvement, as was the case for the two patients who presented exclusively with cardiac manifestations (L150, L165).

The findings of this study also suggest that for patients with clinical involvement of more than one system, the rate of disease progression may vary between different organ systems. This idea is supported by the observation that, within the current sample, none of the patients who died within the first 6 months of life due to severe neuromuscular weakness (scored as N3) were reported to exhibit clinically severe hepatic disease, as defined previously (Figure 3). However, a number of these patients exhibited milder evidence of hepatic disease, such as hepatomegaly (L65, L90, L92, L96, and L149), elevated transaminases (L45, L74, L75, and L93), and/or hepatic fibrosis at autopsy (L72 and L102). One possible explanation for this finding is that the rate of clinical disease progression may differ by organ system, such that, among patients with both hepatic and skeletal muscle involvement at the tissue level, more time may be required for hepatic disease manifestations to become clinically severe. Thus, it may be the case that the patients with the most severe degree of neuromuscular involvement died before sufficient time had elapsed for hepatic disease to progress. Whereas hepatic and neuromuscular disease manifestations, when present, tended to become apparent within the first few years of life, the age at which cardiomyopathy was detected varied widely among the patients represented in this sample, ranging from infancy to the third decade of life. However, the extent to which this finding reflects delays in diagnosis, versus true variation in age at onset, is unclear and requires further study. Overall, the factors that contribute to variation in the timing and severity of disease expression across different organ systems remain poorly understood. Careful, long-term clinical surveillance is therefore warranted in all patients.

The second major conclusion of the current study is that variation in disease severity among patients with GSD IV is better characterized as a continuum rather than discrete categories. Under the subtype-based classification system, patients with predominantly hepatic manifestations are classified into either the more severe “classic hepatic” subtype–typically defined by the need for LT by 5 years of age–or the milder “non-progressive” hepatic subtype (Iijima et al., 2018; Magoulas and El-Hattab, 2019; Ichimoto et al., 2020). Careful analysis of hepatic phenotypes in the current study identified several patients whose outcomes appeared to be intermediate between these subtypes (scored as H2). These included two patients (L21 and L44) who survived beyond 5 years of age without transplant and thus did not meet the definition of the classic hepatic subtype, but later went on to develop severe hepatic complications, including hepatocellular carcinoma in one case, that led to death or LT during the second decade of life. These findings suggest that there is graded variation in the rate of hepatic disease progression in GSD IV, undermining the dichotomization of hepatic disease into just two subtypes.

Neuromuscular disease manifestations may likewise vary along a continuum. The established classification system recognizes the perinatal-congenital neuromuscular subtype–characterized by onset of profound weakness at or before birth, leading to respiratory insufficiency and death during early infancy–as distinct from the juvenile neuromuscular subtype (Szymanska et al., 2018; Magoulas and El-Hattab, 2019). However, the current study identified several patients whose clinical course appeared to be intermediate between these subtypes (L55, L86, L130, L143, L168, L171, and L172). These patients (scored as N2) presented with significant weakness and/or contractures at the time of birth but survived beyond the newborn period and exhibited a more protracted clinical course compared to the most severely-affected patients. Several of these patients have been followed well into childhood or even adolescence, and most were reported to exhibit significant motor delays and/or require the use of assistive mobility devices, such as a walker or wheelchair (L86, L168, and L171). For these patients, their timing of symptom onset was most consistent with the perinatal-congenital subtype while their clinical course more closely approximated the juvenile neuromuscular subtype (Burrow et al., 2006). These findings suggest that the neuromuscular manifestations of GSD IV exist along a continuum with respect to clinical severity and the timing of onset.

Collectively, these findings undermine the view of GSD IV as consisting of discrete, mutually exclusive clinical subtypes. It may instead be more accurate to suggest that GBE deficiency has the potential to cause a spectrum of manifestations across multiple systems, and that each affected individual may exhibit different degrees of hepatic, neuromuscular, and/or cardiac involvement at different points in time. Although the scoring system that was utilized in the current study was designed for descriptive purposes and was not intended for use as a prospective clinical scoring tool, it raises important questions about the merits of adopting an approach to phenotypic classification that accounts for graded variation in disease expression across multiple systems. Far from being merely semantic in nature, these questions have important implications for affected patients. In the clinical setting, an accurate understanding of the GSD IV phenotypic spectrum is needed to counsel affected families, identify appropriate candidates for transplantation, and ensure that patients receive adequate long-term surveillance. Additionally, the way in which phenotypic variation is categorized has implications for research. Given the challenges involved in predicting the likely clinical trajectories of individual patients with GSD IV, characterization of potential prognostic markers, including genotype, represents a key priority for future research. Since the ability to draw accurate conclusions about the prognostic value of a putative marker depends, at least in part, on the quality of the phenotypic data used to infer the relationship, the use of imprecise or inaccurate phenotypic categories could obscure relationships that do exist or lead to the identification of spurious associations.

Although the approach to phenotypic characterization that was utilized in the current study yielded a number of key insights, several important limitations are worth noting. As a systematic review, the current study relied on data extracted from a heterogeneous set of papers and abstracts published over the course of more than 50 years, which varied substantially with respect to the extent of clinical, biochemical, genetic, and/or histological data that was reported. In order to accommodate this heterogeneity, the scoring categories utilized in the current study were deliberately broad, which may have subsumed patients with clinically important differences into the same group. In many cases, clinical information was limited or incomplete, as evidenced by the number of patients for which there was not sufficient data to assign a complete phenotypic score. Although the three systems that were assessed (neuromuscular, hepatic, and cardiac) were selected based on the most commonly-reported clinical findings in the published literature on GSD IV, there may be disease manifestations in other organ systems that were not captured in the current study. Moreover, in the interest of diagnostic accuracy, this analysis was restricted to patients with an enzymatically and/or genetically confirmed GSD IV diagnosis (along with their affected siblings, where applicable) and excluded patients who were diagnosed based exclusively on histopathological findings. Collectively, these considerations underscore the need for prospective, longitudinal natural history studies to catalog phenotypic variation in GSD IV in a more comprehensive and granular manner. With continued advances in research, high-quality phenotypic data from human patients will be needed to validate potential prognostic markers, define clinically important endpoints, and evaluate the effects of novel treatments as they become available. Additionally, the current study did not examine the relationship between histopathological findings and clinical disease severity, which is an area that warrants further investigation. Finally, there is also a need for additional studies to capture the spectrum of phenotypic variation in the adult-onset form of GBE deficiency (APBD), as this was not addressed by the current study.

## 5 Conclusion

The current study systematically characterized phenotypic variation in patients with youth-onset GSD IV based on a comprehensive review of data from all eligible cases in the published literature. Overall, the findings of this study challenge the existence of discrete subtypes and instead support the view that there is continuous variation in the pattern and severity of disease expression in patients with GSD IV. Importantly, the timing of onset and rate of disease progression may differ not just between different patients but also between different organ systems within the same patient, underscoring the need for all patients with GSD IV to receive comprehensive, long-term clinical surveillance. Although this study was limited by its reliance on retrospective, incomplete data, it nonetheless demonstrated the potential value, from both a clinical and research perspective, of adopting an alternative approach to phenotypic classification that accounts for the existence of multisystem involvement and graded variation in disease severity. A better understanding of the full spectrum of phenotypic variation associated with GSD IV may lead to improved outcomes for affected patients.

## Data Availability

The original contributions presented in the study are included in the article/Supplementary Material, further inquiries can be directed to the corresponding author.
