# Supplementary material for: A novel approach to characterize phenotypic variation in GSD IV: Reconceptualizing the clinical continuum
Source: Front Genet. 2022 Sep 13;13:992406. doi: 10.3389/fgene.2022.992406 (PMC9513518; doi:10.3389/fgene.2022.992406)
Supplement: Supplementary file 1 [file DataSheet2.docx]

**Table S1. Literature IDs, Phenotypes, and Original References for Patients with a Confirmed GSD IV Diagnosis.** Patients identified via a systematic search of published references were assigned a unique Literature ID (“Lit ID”). Disease expression was assessed by system (any history of hepatic, neuromuscular, and/or cardiac involvement) based on the criteria detailed in Figure 2. The severity of hepatic and neuromuscular manifestations was further characterized, as detailed in Figure 3, by assigning all patients with available data a neuromuscular score (N score; N0-N3) and hepatic score (H score; H0-H3). The original publication(s) describing each patient were recorded (“Citation” columns) along with the identifier used to describe the patient in the original publication, if applicable. Unless denoted with an asterisk, all patients were diagnosed with GSD IV based on enzymatic confirmation of low GBE activity and/or biallelic GBE1 variants.

* Denotes patients who were diagnosed with GSD IV based on histology (without genetic or enzymatic confirmation) but were included because an affected sibling had an enzymatically- or genotypically-confirmed diagnosis.

*n.s.* = “not scored” due to insufficient information and/or duration of follow-up

|  | Disease Expression by System | | | Phenotypic Score | | References | | | |
| --- | --- | --- | --- | --- | --- | --- | --- | --- | --- |
| **Lit ID** | **Any cardiac?** | **Any neuro-muscular?** | **Any hepatic?** | **N score** | **H score** | **Citation #1**  **(Original ID)** | **Citation #2**  **(Original ID)** | **Citation #3**  **(Original ID)** | **Citation #4**  **(Original ID)** |
| **L7*** | Yes | Yes | Yes | N1 | H3 | Holleman et al., 1966 |  |  |  |
| **L8** | No | Yes | Yes | N1 | H3 | Fernandes and Huijing, 1968 |  |  |  |
| **L9** | Yes | Yes | Yes | N1 | H3 | Reed et al., 1968 | Brown and Brown, 1966  (Patient M.G) |  |  |
| **L10** | *n.s.* | *n.s.* | Yes | *n.s.* | H3 | Howell et al., 1971  (Patient D.H.) |  |  |  |
| **L11** | Yes | Yes | Yes | N1 | H3 | Howell et al., 1971  (Patient T.B.) | McMaster et al., 1979 |  |  |
| **L12** | No | Yes | Yes | N2 | H3 | Zellweger et al., 1972  (Patient D.D.) | Schochet Jr et al., 1970 | Howell et al., 1971  (Patient D.D.) |  |
| **L13** | No | Yes | Yes | N1 | H3 | Ishihara et al., 1975  (Case 2) |  |  |  |
| **L14*** | *n.s.* | *n.s.* | Yes | *n.s.* | H3 | Ishihara et al., 1975  (Case 1) |  |  |  |
| **L15** | *n.s.* | *n.s.* | Yes | *n.s.* | *n.s.* | Bannayan et al., 1976 |  |  |  |
| **L17** | No | Yes | Yes | N1 | *n.s.* | Friedman et al., 1978 |  |  |  |
| **L20** | *n.s.* | No | Yes | N0 | H2 | Guerra et al., 1986 |  |  |  |
| **L21** | No | Yes | Yes | N1 | H2 | Maaswinkel-Mooy et al., 1987 | de Moor et al., 2000 |  |  |
| **L22** | Yes | *n.s.* | *n.s.* | *n.s.* | *n.s.* | Servidei et al., 1987 |  |  |  |
| **L23** | No | No | Yes | N0 | H1 | Greene et al., 1988a | McConkie-Rosell et al., 1996  (Patient A) |  |  |
| **L24** | *n.s.* | Yes | Yes | N1 | H3 | Greene et al., 1988b  (Patient 1) |  |  |  |
| **L25** | No | Yes | Yes | N1 | H3 | Greene et al., 1988b  (Patient 2) |  |  |  |
| **L28** | No | Yes | Yes | *n.s.* | H3 | Selby et al., 1991  (Patient 1) | Selby et al., 1993  (Patient 1) | Starzl et al., 1993  (Patient 1) | Matern et al., 1999  (Case 7) |
| **L29** | No | Yes | Yes | *n.s.* | H3 | Selby et al., 1991  (Patient 2) | Selby et al., 1993  (Patient 2) | Matern et al., 1999  (Case 2) |  |
| **L30** | No | Yes | Yes | *n.s.* | H3 | Selby et al., 1991  (Patient 3) | Selby et al., 1993  (Patient 3) | Matern et al., 1999  (Case 9) |  |
| **L31** | No | Yes | Yes | *n.s.* | H3 | Selby et al., 1991  (Patient 4) | Selby et al., 1993  (Patient 4) | Matern et al., 1999  (Case 11) |  |
| **L32** | No | Yes | Yes | *n.s.* | H3 | Selby et al., 1991  (Patient 5) | Selby et al., 1993  (Patient 5) | Matern et al., 1999  (Case 6) |  |
| **L33** | No | Yes | Yes | *n.s.* | H3 | Selby et al., 1991  (Patient 6) | Selby et al., 1993  (Patient 6) | Starzl et al., 1993  (Patient 2) | Matern et al., 1999  (Case 5) |
| **L34** | No | Yes | Yes | *n.s.* | H3 | Selby et al., 1991  (Patient 7) | Selby et al., 1993  (Patient 7) | Matern et al., 1999  (Case 8) |  |
| **L35** | No | Yes | Yes | N1 | H1 | Reusche et al., 1992  (Patient 1) |  |  |  |
| **L36** | No | Yes | Yes | N1 | H1 | Reusche et al., 1992  (Patient 2) |  |  |  |
| **L37** | *n.s.* | *n.s.* | *n.s.* | *n.s.* | *n.s.* | Penchansky et al., 1992 |  |  |  |
| **L38** | Yes | No | Yes | N0 | H3 | Sokal et al., 1992 |  |  |  |
| **L40** | *n.s.* | Yes | *n.s.* | N3 | *n.s.* | van Noort et al., 1993  (Case 2) | Bruno et al., 2004  (Patient 3) |  |  |
| **L41** | Yes | Yes | Yes | N2 | H2 | Schroder et al., 1993  (Patient 1) | Nase et al., 1995  (Patient 1) |  |  |
| **L42*** | Yes | Yes | *n.s.* | N2 | *n.s.* | Schroder et al., 1993  (Patient 2) | Nase et al., 1995  (Patient 2) |  |  |
| **L43** | Yes | No | Yes | N0 | H3 | Alshak et al., 1994 | Rosenthal et al., 1995 |  |  |
| **L44** | No | No | Yes | N0 | H2 | Dhawan et al., 1994 |  |  |  |
| **L45** | No | Yes | Yes | N3 | H1 | Herrick et al., 1994 |  |  |  |
| **L47** | Yes | Yes | Yes | N3 | H1 | Tang et al., 1994 | Bao et al., 1996  (Patient 1) |  |  |
| **L48** | *n.s.* | *n.s.* | Yes | *n.s.* | H3 | Bao et al., 1996  (Patient 3) |  |  |  |
| **L49** | *n.s.* | *n.s.* | Yes | *n.s.* | H3 | Bao et al., 1996  (Patient 4) |  |  |  |
| **L50** | No | No | Yes | N0 | H1 | McConkie-Rosell et al., 1996  (Patient B) |  |  |  |
| **L51** | No | No | Yes | N0 | H1 | McConkie-Rosell et al., 1996  (Patient C) | Bao et al., 1996  (Patient 2) | Shen et al., 1999  (The proband) |  |
| **L52** | No | No | Yes | N0 | H1 | McConkie-Rosell et al., 1996  (Patient D) |  |  |  |
| **L53** | *n.s.* | No | Yes | N0 | H3 | Lee et al., 1998 |  |  |  |
| **L54** | *n.s.* | Yes | No | N3 | H0 | Alegria et al., 1999 |  |  |  |
| **L55** | No | Yes | Yes | N2 | H3 | Bruno et al., 1999 | Bruno et al., 2004  (Patient 8) |  |  |
| **L56** | *n.s.* | Yes | Yes | N2 | H1 | Chan et al., 1999 |  |  |  |
| **L63** | *n.s.* | *n.s.* | Yes | *n.s.* | H1 | Shen et al., 1999  (the fetus) |  |  |  |
| **L65** | Yes | Yes | Yes | N3 | H1 | Nambu et al., 2003 |  |  |  |
| **L66** | *n.s.* | Yes | No | N3 | H0 | Bruno et al., 2004  (Patient 4) |  |  |  |
| **L67** | *n.s.* | Yes | No | N3 | H0 | Bruno et al., 2004  (Patient 5) |  |  |  |
| **L68** | *n.s.* | Yes | No | N3 | H0 | Bruno et al., 2004  (Patient 6) |  |  |  |
| **L69** | *n.s.* | Yes | No | N1 | H0 | Bruno et al., 2004  (Patient 7) |  |  |  |
| **L70** | Yes | Yes | *n.s.* | N3 | *n.s.* | Giuffre et al., 2004  (Patient 2) |  |  |  |
| **L71*** | Yes | Yes | No | N3 | H0 | Giuffre et al., 2004  (Patient 1) |  |  |  |
| **L72** | Yes | Yes | No | N3 | H0 | Janecke et al., 2004 | Konstantinidou et al., 2008  (Patient 2) |  |  |
| **L73** | No | Yes | Yes | N1 | *n.s.* | Kotb et al., 2004  (Case 9) |  |  |  |
| **L74** | Yes | Yes | Yes | N3 | H1 | Maruyama et al., 2004  (Case 1) |  |  |  |
| **L75** | Yes | Yes | Yes | N3 | H1 | Maruyama et al., 2004  (Case 2) |  |  |  |
| **L77** | No | Yes | No | N3 | H0 | Tay et al., 2004  (Patient 1) | Akman et al., 2006  (Family 2, propositus) |  |  |
| **L78** | No | Yes | *n.s.* | N3 | *n.s.* | Tay et al., 2004  (Patient 2) |  |  |  |
| **L83** | *n.s.* | *n.s.* | *n.s.* | *n.s.* | *n.s.* | Akman et al., 2006  (Family 1, fetus) | Konstantinidou et al., 2008 | Destouni et al., 2010 |  |
| **L84** | *n.s.* | Yes | *n.s.* | N3 | *n.s.* | Akman et al., 2006  (Family 1, propositus) | Konstantinidou et al., 2008 | Destouni et al., 2010 |  |
| **L86** | No | Yes | Yes | N2 | H1 | Burrow et al., 2006 |  |  |  |
| **L87** | No | Yes | No | N3 | H0 | Assereto et al., 2007  (Patient 1) |  |  |  |
| **L88** | No | Yes | No | N3 | H0 | Assereto et al., 2007  (Patient 2) |  |  |  |
| **L89** | Yes | Yes | Yes | N1 | H2 | Eminoglu et al., 2008 |  |  |  |
| **L90** | Yes | Yes | Yes | N3 | H1 | Nolte et al., 2008  (Case 1) |  |  |  |
| **L91** | Yes | Yes | *n.s.* | N3 | *n.s.* | Nolte et al., 2008  (Case 2) |  |  |  |
| **L92** | Yes | Yes | Yes | N3 | H1 | Nolte et al., 2008  (Case 3) |  |  |  |
| **L93** | Yes | Yes | Yes | N3 | H1 | Raju et al., 2008 |  |  |  |
| **L94** | Yes | No | Yes | N0 | H3 | Ban et al., 2009 |  |  |  |
| **L95** | No | Yes | No | N3 | H0 | Jimenez-Mallebrera et al., 2009 |  |  |  |
| **L96** | Yes | Yes | Yes | N3 | H1 | Lamperti et al., 2009 | Marchesi et al., 2009 |  |  |
| **L98** | No | Yes | No | N2 | H0 | Fernandez et al., 2010 |  |  |  |
| **L99** | *n.s.* | Yes | Yes | N1 | *n.s.* | Li et al., 2010  (Patient 1) |  |  |  |
| **L100** | *n.s.* | *n.s.* | *n.s.* | *n.s.* | *n.s.* | Li et al., 2010  (Patient 2) |  |  |  |
| **L101** | *n.s.* | Yes | Yes | *n.s.* | *n.s.* | Li et al., 2010  (Patient 3) |  |  |  |
| **L102** | Yes | Yes | *n.s.* | N3 | *n.s.* | Taratuto et al., 2010 |  |  |  |
| **L103** | Yes | Yes | Yes | N1 | H3 | Willot et al., 2010  (Case 2) |  |  |  |
| **L106** | Yes | Yes | Yes | N1 | H3 | Mizuochi et al., 2011 |  |  |  |
| **L108** | Yes | *n.s.* | Yes | *n.s.* | *n.s.* | Aksu et al., 2012  (Case 1) |  |  |  |
| **L109** | Yes | *n.s.* | Yes | *n.s.* | H1 | Aksu et al., 2012  (Case 2) |  |  |  |
| **L110** | No | Yes | *n.s.* | N3 | *n.s.* | Escobar et al., 2012 |  |  |  |
| **L111** | Yes | Yes | *n.s.* | N3 | *n.s.* | Li et al., 2012  (Case 1) |  |  |  |
| **L112** | Yes | Yes | *n.s.* | N3 | *n.s.* | Li et al., 2012  (Case 2) |  |  |  |
| **L113** | Yes | Yes | Yes | N1 | H3 | Magoulas et al., 2012  (“The infant girl”) |  |  |  |
| **L114** | Yes | *n.s.* | Yes | *n.s.* | H3 | Magoulas et al., 2012  (“Second child”) |  |  |  |
| **L121** | No | No | Yes | N0 | H3 | Santra et al., 2013 | Sreekantam et al., 2020 |  |  |
| **L122** | No | No | Yes | N0 | *n.s.* | Hizarcioglu-Gulsen et al., 2014 |  |  |  |
| **L125** | *n.s.* | Yes | Yes | N1 | H1 | Ersoy and Onal, 2015 |  |  |  |
| **L130** | No | Yes | No | N2 | H0 | Malfatti et al., 2016 |  |  |  |
| **L131** | No | Yes | Yes | *n.s.* | H1 | Said et al., 2016 |  |  |  |
| **L132** | Yes | Yes | No | N3 | H0 | Schanzer et al., 2016 |  |  |  |
| **L133** | No | Yes | Yes | N2 | H3 | Forny et al., 2017 | Forny et al., 2021 |  |  |
| **L136** | *n.s.* | Yes | Yes | N1 | H3 | Stonko and Crook, 2017 |  |  |  |
| **L137** | No | No | Yes | N0 | H3 | Choi et al., 2018 |  |  |  |
| **L138** | No | No | Yes | N0 | H1 | Iijima et al., 2018 |  |  |  |
| **L139** | No | *n.s.* | Yes | *n.s.* | H3 | Pan et al., 2018 |  |  |  |
| **L140** | No | Yes | No | N3 | H0 | Sandhu et al., 2019 |  |  |  |
| **L141** | No | Yes | Yes | N1 | H1 | Schene et al., 2019  (Patient 1) |  |  |  |
| **L142** | No | Yes | Yes | N1 | H1 | Schene et al., 2019  (Patient 2) | Derks et al., 2021  (Patient 7) |  |  |
| **L143** | Yes | Yes | Yes | N2 | H3 | Szymanska et al., 2018  (Patient 1) | Derks et al., 2021  (Patient 12) |  |  |
| **L144** | *n.s.* | No | Yes | N0 | H1 | Szymanska et al., 2018  (Patient 2) | Derks et al., 2021  (Patient 13) |  |  |
| **L145** | Yes | No | No | N0 | H0 | Szymanska et al., 2018  (Patient 3) | Derks et al., 2021  (Patient 14) |  |  |
| **L146** | No | Yes | No | N2 | H0 | Walter et al., 2018 |  |  |  |
| **L147** | *n.s.* | Yes | *n.s.* | N3 | *n.s.* | Yu et al., 2018 |  |  |  |
| **L149** | Yes | Yes | Yes | N3 | H1 | Massey and Dannaway, 2019 |  |  |  |
| **L150** | Yes | No | No | N0 | H0 | Ndugga-Kabuye et al., 2019 |  |  |  |
| **L152** | No | Yes | No | N3 | H0 | Radhakrishnan et al., 2019  (F2-II:2) |  |  |  |
| **L153** | *n.s.* | Yes | *n.s.* | N3 | *n.s.* | Radhakrishnan et al., 2019  (F2-II:3) |  |  |  |
| **L155** | *n.s.* | Yes | *n.s.* | N3 | *n.s.* | Butler et al., 2020 |  |  |  |
| **L156** | *n.s.* | *n.s.* | Yes | *n.s.* | *n.s.* | Cakar et al., 2020  (Patient 5) |  |  |  |
| **L157** | No | No | Yes | N0 | H1 | Ichimoto et al., 2020  (Case 2) |  |  |  |
| **L158** | No | No | Yes | N0 | H1 | Ichimoto et al., 2020  (Case 1) |  |  |  |
| **L161** | *n.s.* | *n.s.* | Yes | *n.s.* | *n.s.* | Wei et al., 2020 |  |  |  |
| **L162** | *n.s.* | No | Yes | N0 | H1 | Ying et al., 2020  (Patient 28) |  |  |  |
| **L163** | No | Yes | Yes | N1 | H3 | Derks et al., 2021  (Patient 1) |  |  |  |
| **L164** | No | Yes | Yes | N1 | H1 | Derks et al., 2021  (Patient 2) |  |  |  |
| **L165** | No | Yes | No | N1 | H0 | Derks et al., 2021  (Patient 3) |  |  |  |
| **L166** | No | No | Yes | N0 | H3 | Derks et al., 2021  (Patient 4) |  |  |  |
| **L167** | No | No | Yes | N0 | H2 | Derks et al., 2021  (Patient 5) |  |  |  |
| **L168** | No | Yes | Yes | N2 | H3 | Derks et al., 2021  (Patient 6) |  |  |  |
| **L169** | No | Yes | Yes | N1 | H1 | Derks et al., 2021  (Patient 8) |  |  |  |
| **L170** | No | No | Yes | N0 | H1 | Derks et al., 2021  (Patient 9) |  |  |  |
| **L171** | No | Yes | Yes | N2 | H1 | Derks et al., 2021  (Patient 10) |  |  |  |
| **L172** | No | Yes | Yes | N2 | H2 | Derks et al., 2021  (Patient 11) |  |  |  |
| **L173** | No | Yes | Yes | N2 | *n.s.* | Derks et al., 2021  (Patient 15) |  |  |  |
| **L174** | No | Yes | Yes | N1 | H3 | Ersoy et al., 2021  (P13) |  |  |  |
| **L175** | No | Yes | Yes | *n.s.* | H3 | Ersoy et al., 2021  (P14) |  |  |  |
| **L176** | *n.s.* | *n.s.* | Yes | *n.s.* | H3 | Beyzaei et al., 2022  (P7) | Beyzaei et al., 2021  (Patient 6) |  |  |
| **L178** | *n.s.* | *n.s.* | *n.s.* | *n.s.* | *n.s.* | Demir et al., 2022 |  |  |  |

**Table S2. GSD IV Patients Reported as Deceased In-Utero.** Patients with an enzymatically- or genetically-confirmed GSD IV diagnosis who died in-utero were included in the current study but were not assigned a phenotypic score. The original publication(s) describing each patient were recorded (“Citation” columns) along with the identifier used to describe the patient in the original publication, if applicable.

| **Lit ID** | **Citation #1**  **(Original ID)** | **Citation #2**  **(Original ID)** |
| --- | --- | --- |
| L39 | van Noort et al., 1993 (Case 1) | Bruno et al., 2004 (Patient 2) |
| L57 | Cox et al., 1999 (Sib 3) |  |
| L58 | Cox et al., 1999 (Sib 1) |  |
| L59 | Cox et al., 1999 (Sib 2) |  |
| L79 | L'Hermine-Coulomb et al., 2005 (Second pregnancy) |  |
| L80 | L'Hermine-Coulomb et al., 2005 (Third pregnancy) |  |
| L81 | L'Hermine-Coulomb et al., 2005 (First pregnancy) |  |
| L85 | Akman et al., 2006 (Family 2, fetus) |  |
| L118 | Ravenscroft et al., 2013 (II:1) |  |
| L119 | Ravenscroft et al., 2013 (II:2) |  |
| L120 | Ravenscroft et al., 2013 (II:3) |  |
| L123 | Alamillo et al., 2015 |  |
| L127 | Bendroth-Asmussen et al., 2016 (“the second pregnancy”) |  |
| L128 | Bendroth-Asmussen et al., 2016 (“the first pregnancy”) |  |
| L129 | Dainese et al., 2016 |  |
| L151 | Radhakrishnan et al., 2019 (F1-II:2) |  |
| L154 | Radhakrishnan et al., 2019 (F3-II:3) |  |
| L179 | Marangoni et al., 2022 (R27) |  |

**Table S3. Literature IDs and Original References of Patients not Meeting Full Inclusion Criteria.** Patients in whom the diagnosis of GSD IV was not reported to have been confirmed by enzymology or genotype were assigned a Literature ID (“Lit ID”) but were not assigned a phenotypic score. The original publication(s) describing each patient were recorded (“Citation” columns) along with the identifier used to describe the patient in the original publication, if applicable.

| **Lit ID** | **Citation #1**  **(Original ID)** | **Citation #2**  **(Original ID)** |
| --- | --- | --- |
| L1 | Andersen, 1952 | Andersen, 1956 |
| L2 | Craig and Uzman, 1958 (Case 1) |  |
| L3 | Craig and Uzman, 1958 (Case 2) |  |
| L4 | Craig and Uzman, 1958 (Case 3) |  |
| L5 | Sidbury Jr et al., 1962 |  |
| L6 | Mortimer, 1965 | Levin et al., 1968 |
| L16 | Cantin et al., 1976 |  |
| L18 | Kalra et al., 1980 |  |
| L19 | Heyman, 1985 |  |
| L26 | Tunon et al., 1988 |  |
| L27 | Santonja et al., 1990 |  |
| L46 | Sarkar et al., 1994 |  |
| L60 | Matern et al., 1999 (Case 4) |  |
| L61 | Matern et al., 1999 (Case 10) |  |
| L62 | Matern et al., 1999 (Case 12) |  |
| L64 | Sahoo et al., 2002 |  |
| L76 | Michelakakis et al., 2004 |  |
| L82 | Morioka et al., 2005 (Table 3, “GSD Type IV Boy”) |  |
| L97 | Onal et al., 2009 |  |
| L104 | Willot et al., 2010 (Case 1) |  |
| L105 | Fowkes, 2011 |  |
| L107 | Romano et al., 2011 (Patient no. 7) |  |
| L115 | Sluiter et al., 2012 (Table 3, Row 10) |  |
| L116 | Sluiter et al., 2012 (Table 3, Row 11) |  |
| L117 | Sluiter et al., 2012 (Table 3, Row 12) |  |
| L124 | Bostancı et al., 2015 |  |
| L126 | Kakkar et al., 2015 |  |
| L134 | Gurrieri et al., 2017 (Patient 16) |  |
| L135 | Soto et al., 2017 |  |
| L148 | Cardoso M.T., 2019 |  |
| L159 | Lyo et al., 2020 |  |
| L160 | Mastrocola et al., 2020 |  |
| L177 | Beyzaei et al., 2022 (P2) |  |

**Table S4. Hepatic and Neuromuscular Disease Manifestations in GSD IV.** The frequency with which select hepatic and neuromuscular disease manifestations were reported is shown. For the “overall frequency” calculations, the denominator included all patients with any hepatic involvement (for hepatic manifestations) or any neuromuscular involvement (for neuromuscular manifestations).

^1^ Development was not assessed for the N3 patients who were deceased within the first 6 months of life. The total frequency is calculated out of 51 eligible patients.

|  | **Hepatic Manifestation** | **Overall frequency**  **(n = 90)** |  | **Neuromuscular Manifestation** | **Overall frequency**  **(n = 88)** |  |
| --- | --- | --- | --- | --- | --- | --- |
|  | Hepatomegaly | 82.2% |  | Hypotonia | 68.2% |  |
|  |  |  |  |  |  |  |
|  |  |  |  |  |  |  |
|  | ↑ALT | 63.3% |  | Abnormal motor development^1^ | 56.9% |  |
|  |  |  |  |  |  |  |
|  |  |  |  |  |  |  |
|  | Splenomegaly | 65.6% |  | Hyporeflexia | 31.8% |  |
|  |  |  |  |  |  |  |
|  |  |  |  |  |  |  |
|  | Synthetic Dysfunction  (↑PT/INR or ↓Albumin) | 30.0% |  | Contractures or arthrogryposis | 31.8% |  |
|  |  |  |  |  |  |  |
|  |  |  |  |  |  |  |
|  | Ascites | 28.9% |  | ↑CK | 25.0% |  |
|  |  |  |  |  |  |  |
|  |  |  |  |  |  |  |
|  | Jaundice or ↑Bilirubin | 24.4% |  | Muscle atrophy or hypotrophy | 23.9% |  |
|  |  |  |  |  |  |  |
|  |  |  |  |  |  |  |
|  | Hypoglycemia | 17.8% |  |  | |  |
|  | Varices | 17.8% |  |  |  |  |

**References**

Akman, H.O., Karadimas, C., Gyftodimou, Y., Grigoriadou, M., Kokotas, H., Konstantinidou, A., et al. (2006). Prenatal diagnosis of glycogen storage disease type IV. *Prenat Diagn* 26(10)**,** 951-955. doi: 10.1002/pd.1533.

Aksu, T., Colak, A., and Tufekcioglu, O. (2012). Cardiac Involvement in Glycogen Storage Disease Type IV: Two Cases and the Two Ends of a Spectrum. *Case Rep Med* 2012**,** 764286. doi: 10.1155/2012/764286.

Alamillo, C.L., Powis, Z., Farwell, K., Shahmirzadi, L., Weltmer, E.C., Turocy, J., et al. (2015). Exome sequencing positively identified relevant alterations in more than half of cases with an indication of prenatal ultrasound anomalies. *Prenat Diagn* 35(11)**,** 1073-1078. doi: 10.1002/pd.4648.

Alegria, A., Martins, E., Dias, M., Cunha, A., Cardoso, M.L., and Maire, I. (1999). Glycogen storage disease type IV presenting as hydrops fetalis. *J Inherit Metab Dis* 22(3)**,** 330-332. doi: 10.1023/a:1005568507267.

Alshak, N.S., Cocjin, J., Podesta, L., van de Velde, R., Makowka, L., Rosenthal, P., et al. (1994). Hepatocellular adenoma in glycogen storage disease type IV. *Arch Pathol Lab Med* 118(1)**,** 88-91.

Andersen, D. (1956). Familial cirrhosis of the liver with storage of abnormal glycogen. *Lab. Invest.* 5**,** 11-20.

Andersen, D.H. (1952). Studies on glycogen disease with report of a case in which the glycogen was abnormal. *Carbohydrate metabolism***,** 28.

Assereto, S., van Diggelen, O.P., Diogo, L., Morava, E., Cassandrini, D., Carreira, I., et al. (2007). Null mutations and lethal congenital form of glycogen storage disease type IV. *Biochem Biophys Res Commun* 361(2)**,** 445-450. doi: 10.1016/j.bbrc.2007.07.074.

Ban, H.R., Kim, K.M., Jang, J.Y., Kim, G.H., You, H.W., Kim, K., et al. (2009). Living Donor Liver Transplantation in a Korean Child with Glycogen Storage Disease Type IV and a GBE1 Mutation. *Gut Liver* 3(1)**,** 60-63. doi: 10.5009/gnl.2009.3.1.60.

Bannayan, G.A., Dean, W.J., and Howell, R.R. (1976). Type IV glycogen-storage disease. Light-microscopic, electron-microscopic, and enzymatic study. *Am J Clin Pathol* 66(4)**,** 702-709. doi: 10.1093/ajcp/66.4.702.

Bao, Y., Kishnani, P., Wu, J.Y., and Chen, Y.T. (1996). Hepatic and neuromuscular forms of glycogen storage disease type IV caused by mutations in the same glycogen-branching enzyme gene. *J Clin Invest* 97(4)**,** 941-948. doi: 10.1172/JCI118517.

Bendroth-Asmussen, L., Aksglaede, L., Gernow, A.B., and Lund, A.M. (2016). Glycogen Storage Disease Type IV: A Case With Histopathologic Findings in First-Trimester Placental Tissue. *Int J Gynecol Pathol* 35(1)**,** 38-40. doi: 10.1097/PGP.0000000000000214.

Beyzaei, Z., Ezgu, F., Geramizadeh, B., Imanieh, M.H., Haghighat, M., Dehghani, S.M., et al. (2021). Clinical and genetic spectrum of glycogen storage disease in Iranian population using targeted gene sequencing. *Scientific reports* 11(1)**,** 1-9.

Beyzaei, Z., Shamsaeefar, A., Kazemi, K., Nikeghbalian, S., Bahador, A., Dehghani, M., et al. (2022). Liver transplantation in glycogen storage disease: a single-center experience. *Orphanet J Rare Dis* 17(1)**,** 127. doi: 10.1186/s13023-022-02284-y.

Bostancı, E.B., Öter, V., Özer, İ., Turhan, N., and Akoğlu, M. (2015). "Living Donor Liver Transplantation For Glycogen Storage Disease Type IV With Hepatocellular Carcinoma; A Case Report", in: *24th Conference of APASL.*).

Brown, B.I., and Brown, D.H. (1966). Lack of an alpha-1, 4-glucan: alpha-1, 4-glucan 6-glycosyl transferase in a case of type IV glycogenosis. *Proceedings of the National Academy of Sciences of the United States of America* 56(2)**,** 725.

Bruno, C., DiRocco, M., Lamba, L.D., Bado, M., Marino, C., Tsujino, S., et al. (1999). A novel missense mutation in the glycogen branching enzyme gene in a child with myopathy and hepatopathy. *Neuromuscul Disord* 9(6-7)**,** 403-407. doi: 10.1016/s0960-8966(99)00040-1.

Bruno, C., van Diggelen, O.P., Cassandrini, D., Gimpelev, M., Giuffre, B., Donati, M.A., et al. (2004). Clinical and genetic heterogeneity of branching enzyme deficiency (glycogenosis type IV). *Neurology* 63(6)**,** 1053-1058. doi: 10.1212/01.wnl.0000138429.11433.0d.

Burrow, T.A., Hopkin, R.J., Bove, K.E., Miles, L., Wong, B.L., Choudhary, A., et al. (2006). Non-lethal congenital hypotonia due to glycogen storage disease type IV. *Am J Med Genet A* 140(8)**,** 878-882. doi: 10.1002/ajmg.a.31166.

Butler, D.C., Glen, W.B., Jr., Schandl, C., and Phillips, A. (2020). Glycogen Storage Disease Type IV Diagnosed at Fetal Autopsy. *Pediatr Dev Pathol* 23(4)**,** 301-305. doi: 10.1177/1093526619890224.

Cakar, N.E., Gezdirici, A., Topuz, H.S., and Onal, H. (2020). Novel variants in Turkish patients with glycogen storage disease. *Pediatr Int* 62(10)**,** 1145-1150. doi: 10.1111/ped.14286.

Cantin, M., Brochu, P., Turgeon-Knaack, C., Berdnikoff, G., Simard, P., and Morin, C. (1976). Rectal biopsy in type 4 glycogenosis. An ultrastructural cytochemical study. *Arch Pathol Lab Med* 100(8)**,** 422-426.

Cardoso M.T., C.P.C., Pintalhao M., Abreu P., Vasconcelos M., Leao M., Rodrigues E., Leao E (2019). Glycogen Storage Disease Type IV: A manifesting heterozygous through a mild late onset phenotype with hepatic and myopathic presentation. *Journal of Inherited Metabolic Disease* 42 Supplement 1(311).

Chan, Y.J., Lin, S.P., and Chen, B.F. (1999). Glycogen storage disease type IV: a case report. *Zhonghua Yi Xue Za Zhi (Taipei)* 62(10)**,** 743-747.

Choi, S.Y., Kang, B., Choe, J.Y., Lee, Y., Jang, H.J., Park, H.D., et al. (2018). A Case of Glycogen Storage Disease IV with Rare Homozygous Mutations in the Glycogen Branching Enzyme Gene. *Pediatr Gastroenterol Hepatol Nutr* 21(4)**,** 365-368. doi: 10.5223/pghn.2018.21.4.365.

Cox, P.M., Brueton, L.A., Murphy, K.W., Worthington, V.C., Bjelogrlic, P., Lazda, E.J., et al. (1999). Early-onset fetal hydrops and muscle degeneration in siblings due to a novel variant of type IV glycogenosis. *Am J Med Genet* 86(2)**,** 187-193. doi: 10.1002/(sici)1096-8628(19990910)86:2<187::aid-ajmg20>3.0.co;2-7.

Craig, J.M., and Uzman, L.L. (1958). A familial metabolic disorder with storage of an unusual polysaccharide complex. *Pediatrics* 22(1, Part 1)**,** 20-32.

Dainese, L., Adam, N., Boudjemaa, S., Hadid, K., Rosenblatt, J., Jouannic, J.M., et al. (2016). Glycogen Storage Disease Type IV and Early Implantation Defect: Early Trophoblastic Involvement Associated with a New GBE1 Mutation. *Pediatr Dev Pathol* 19(6)**,** 512-515. doi: 10.2350/14-09-1557-CR.1.

de Moor, R.A., Schweizer, J.J., van Hoek, B., Wasser, M., Vink, R., and Maaswinkel-Mooy, P.D. (2000). Hepatocellular carcinoma in glycogen storage disease type IV. *Arch Dis Child* 82(6)**,** 479-480. doi: 10.1136/adc.82.6.479.

Demir, B.K., Kanik, A., Kose, M., Hismi, B.O., and Baran, M. (2022). A surprising cause of proteinuria: Answers. *Pediatr Nephrol* 37(5)**,** 1033-1039. doi: 10.1007/s00467-021-05363-7.

Derks, T.G.J., Peeks, F., de Boer, F., Fokkert-Wilts, M., van der Doef, H.P.J., van den Heuvel, M.C., et al. (2021). The potential of dietary treatment in patients with glycogen storage disease type IV. *J Inherit Metab Dis* 44(3)**,** 693-704. doi: 10.1002/jimd.12339.

Destouni, A., Vrettou, C., Traeger-Synodinos, J., Davies, S., Mastrominas, M., and Kanavakis, E. (2010). PGD for glycogen storage disease type IV: birth of healthy twins following successful clinical application of a mutation-specific protocol. *Prenat Diagn* 30(2)**,** 180-182. doi: 10.1002/pd.2435.

Dhawan, A., Tan, K.C., Portmann, B., and Mowat, A.P. (1994). Glycogenosis type IV: liver transplant at 12 years. *Arch Dis Child* 71(5)**,** 450-451. doi: 10.1136/adc.71.5.450.

Eminoglu, T.F., Tumer, L., Okur, I., Olgunturk, R., Hasanoglu, A., Gonul, II, et al. (2008). Multisystem involvement in a patient due to accumulation of amylopectin-like material with diminished branching enzyme activity. *J Inherit Metab Dis* 31 Suppl 2**,** S255-259. doi: 10.1007/s10545-008-0819-8.

Ersoy, M., and Onal, Z. (2015). A novel mutation of the GBE1 gene in a patient with the non-progressive hepatic form of type IV glycogen storage disease. *Journal of Inherited Metabolic Disease* 38(Suppl 1)**,** S183.

Ersoy, M., Uyanik, B., and Gedikbasi, A. (2021). Evaluation of Glycogen Storage Patients: Report of Twelve Novel Variants and New Clinical Findings in a Turkish Population. *Genes (Basel)* 12(12). doi: 10.3390/genes12121987.

Escobar, L.F., Wagner, S., Tucker, M., and Wareham, J. (2012). Neonatal presentation of lethal neuromuscular glycogen storage disease type IV. *J Perinatol* 32(10)**,** 810-813. doi: 10.1038/jp.2011.178.

Fernandes, J., and Huijing, F. (1968). Branching enzyme-deficiency glycogenosis: studies in therapy. *Arch Dis Child* 43(229)**,** 347-352. doi: 10.1136/adc.43.229.347.

Fernandez, C., Halbert, C., De Paula, A.M., Lacroze, V., Froissart, R., Figarella-Branger, D., et al. (2010). Non-lethal neonatal neuromuscular variant of glycogenosis type IV with novel GBE1 mutations. *Muscle Nerve* 41(2)**,** 269-271. doi: 10.1002/mus.21499.

Forny, P., Buerer, C., Truffer-Richard, B., Schumann, A., Hackenberg, A., Buehr, P., et al. (Year). "Improvement of gross motor skills in a GSD-IV patient after liver transplantation", in: *Swiss Medical Weekly*: MH Swiss Medical Publishers Ltd Farnsburgerstr 8, Ch-4132 Muttenz, Switzerland), 26S-27S.

Forny, P., Burda, P., Bode, P., and Rohrbach, M. (2021). Is serum biotinidase enzyme activity a potential marker of perturbed glucose and lipid metabolism? *JIMD Rep* 57(1)**,** 58-66. doi: 10.1002/jmd2.12168.

Fowkes, M. (Year). "Brain Abnormalities Associated With Glycogen Storage Disease, Type IV (Glycogen Branching Enzyme Deficiency), A Case Report", in: *Journal Of Neuropathology and Experimental Neurology*: Lippincott Williams & Wilkins 530 Walnut St, Philadelphia, PA 19106-3621 USA), 546-547.

Friedman, D.J., Lane, A.B., Katz, S., and Jenkins, T. (1978). Glycogen storage disease type IV diagnosed biochemically. A case report. *S Afr Med J* 54(7)**,** 289-291.

Giuffre, B., Parini, R., Rizzuti, T., Morandi, L., van Diggelen, O.P., Bruno, C., et al. (2004). Severe neonatal onset of glycogenosis type IV: clinical and laboratory findings leading to diagnosis in two siblings. *J Inherit Metab Dis* 27(5)**,** 609-619. doi: 10.1023/b:boli.0000042980.45692.bb.

Greene, H.L., Brown, B.I., McClenathan, D.T., Agostini, R.M., Jr., and Taylor, S.R. (1988a). A new variant of type IV glycogenosis: deficiency of branching enzyme activity without apparent progressive liver disease. *Hepatology* 8(2)**,** 302-306. doi: 10.1002/hep.1840080219.

Greene, H.L., Ghishan, F.K., Brown, B., McClenathan, D.T., and Freese, D. (1988b). Hypoglycemia in type IV glycogenosis: hepatic improvement in two patients with nutritional management. *J Pediatr* 112(1)**,** 55-58. doi: 10.1016/s0022-3476(88)80121-5.

Guerra, A.S., van Diggelen, O.P., Carneiro, F., Tsou, R.M., Simoes, S., and Santos, N.T. (1986). A juvenile variant of glycogenosis IV (Andersen disease). *Eur J Pediatr* 145(3)**,** 179-181. doi: 10.1007/BF00446059.

Gurrieri, C., Sprung, J., Weingarten, T.N., and Warner, M.E. (2017). Patients with glycogen storage diseases undergoing anesthesia: a case series. *BMC Anesthesiol* 17(1)**,** 134. doi: 10.1186/s12871-017-0428-x.

Herrick, M.K., Twiss, J.L., Vladutiu, G.D., Glasscock, G.F., and Horoupian, D.S. (1994). Concomitant branching enzyme and phosphorylase deficiencies. An unusual glycogenosis with extensive neuronal polyglucosan storage. *J Neuropathol Exp Neurol* 53(3)**,** 239-246. doi: 10.1097/00005072-199405000-00004.

Heyman, S. (1985). Liver-spleen scintigraphy in glycogen storage disease (glycogenoses). *Clin Nucl Med* 10(12)**,** 839-843. doi: 10.1097/00003072-198512000-00002.

Hizarcioglu-Gulsen, H., Yuce, A., Akcoren, Z., Berberoglu-Ates, B., Aydemir, Y., Sag, E., et al. (2014). A Rare Cause of Elevated Chitotriosidase Activity: Glycogen Storage Disease Type IV. *JIMD Rep* 17**,** 63-66. doi: 10.1007/8904_2014_335.

Holleman, L., Van der Haar, J., and de Vaan, G. (1966). Type IV glycogenosis. *Laboratory investigation; a journal of technical methods and pathology* 15(1 Pt 2)**,** 357-367.

Howell, R.R., Kaback, M.M., and Brown, B.I. (1971). Type IV glycogen storage disease: branching enzyme deficiency in skin fibroblasts and possible heterozygote detection. *J Pediatr* 78(4)**,** 638-642. doi: 10.1016/s0022-3476(71)80466-3.

Ichimoto, K., Fujisawa, T., Shimura, M., Fushimi, T., Tajika, M., Matsunaga, A., et al. (2020). Two cases of a non-progressive hepatic form of glycogen storage disease type IV with atypical liver pathology. *Mol Genet Metab Rep* 24**,** 100601. doi: 10.1016/j.ymgmr.2020.100601.

Iijima, H., Iwano, R., Tanaka, Y., Muroya, K., Fukuda, T., Sugie, H., et al. (2018). Analysis of GBE1 mutations via protein expression studies in glycogen storage disease type IV: A report on a non-progressive form with a literature review. *Mol Genet Metab Rep* 17**,** 31-37. doi: 10.1016/j.ymgmr.2018.09.001.

Ishihara, T., Uchino, F., Adachi, H., Takahashi, M., Watanabe, S., Tsunetoshi, S., et al. (1975). Type IV glycogenosis - a study of two cases. *Acta Pathol Jpn* 25(5)**,** 613-633. doi: 10.1111/j.1440-1827.1975.tb01995.x.

Janecke, A.R., Dertinger, S., Ketelsen, U.P., Bereuter, L., Simma, B., Muller, T., et al. (2004). Neonatal type IV glycogen storage disease associated with "null" mutations in glycogen branching enzyme 1. *J Pediatr* 145(5)**,** 705-709. doi: 10.1016/j.jpeds.2004.07.024.

Jimenez-Mallebrera, C., Nascimento, A., Cusi, V., Corbera, J.R., Rolland, M.O., Froissart, R., et al. (2009). Glycogen branching enzyme deficiency in an infant with severe congenital hypotonia: an emerging diagnosis of muscle weakness in the perinatal period. *Histopathology* 54(6)**,** 765-768. doi: 10.1111/j.1365-2559.2009.03281.x.

Kakkar, A., Sharma, M.C., Nambirajan, A., Sarkar, C., Suri, V., and Gulati, S. (2015). Glycogen storage disorder due to glycogen branching enzyme (GBE) deficiency: a diagnostic dilemma. *Ultrastructural Pathology* 39(4)**,** 293-297.

Kalra, V., Arya, L.S., and Nayak, N.C. (1980). Glycogen storage disease (Type IV): a familial cirrhosis diagnosed by electron microscopy (case report). *Indian Pediatr* 17(7)**,** 625-627.

Konstantinidou, A.E., Anninos, H., Dertinger, S., Nonni, A., Petersen, M., Karadimas, C., et al. (2008). Placental involvement in glycogen storage disease type IV. *Placenta* 29(4)**,** 378-381. doi: <https://dx.doi.org/10.1016/j.placenta.2008.01.005>.

Kotb, M.A., Abdallah, H.K., and Kotb, A. (2004). Liver glycogenoses: are they a possible cause of polyneuropathy? A cross-sectional study. *Journal of tropical pediatrics* 50(4)**,** 196-202.

L'Hermine-Coulomb, A., Beuzen, F., Bouvier, R., Rolland, M.O., Froissart, R., Menez, F., et al. (2005). Fetal type IV glycogen storage disease: clinical, enzymatic, and genetic data of a pure muscular form with variable and early antenatal manifestations in the same family. *Am J Med Genet A* 139A(2)**,** 118-122. doi: 10.1002/ajmg.a.30945.

Lamperti, C., Salani, S., Lucchiari, S., Bordoni, A., Ripolone, M., Fagiolari, G., et al. (2009). Neuropathological study of skeletal muscle, heart, liver, and brain in a neonatal form of glycogen storage disease type IV associated with a new mutation in GBE1 gene. *J Inherit Metab Dis* 32 Suppl 1**,** S161-168. doi: 10.1007/s10545-009-1134-8.

Lee, K.Y., Seo, K.H., Lee, H.K., and Kim, J.W. (1998). Glycogen storage disease type IV: a case report. *J Korean Med Sci* 13(2)**,** 211-214. doi: 10.3346/jkms.1998.13.2.211.

Levin, B., Burgess, E., and Mortimer, P. (1968). Glycogen storage disease type IV, amylopectinosis. *Archives of disease in childhood* 43(231)**,** 548.

Li, S.C., Chen, C.M., Goldstein, J.L., Wu, J.Y., Lemyre, E., Burrow, T.A., et al. (2010). Glycogen storage disease type IV: novel mutations and molecular characterization of a heterogeneous disorder. *J Inherit Metab Dis* 33 Suppl 3**,** S83-90. doi: 10.1007/s10545-009-9026-5.

Li, S.C., Hwu, W.L., Lin, J.L., Bali, D.S., Yang, C., Chu, S.M., et al. (2012). Association of the congenital neuromuscular form of glycogen storage disease type IV with a large deletion and recurrent frameshift mutation. *J Child Neurol* 27(2)**,** 204-208. doi: 10.1177/0883073811415107.

Lyo, S., Miles, J., Meisner, J., and Guelfguat, M. (2020). Case report: adult-onset manifesting heterozygous glycogen storage disease type IV with dilated cardiomyopathy and absent late gadolinium enhancement on cardiac magnetic resonance imaging. *Eur Heart J Case Rep* 4(3)**,** 1-6. doi: 10.1093/ehjcr/ytaa078.

Maaswinkel-Mooy, P.D., Poorthuis, B.J., van Gelderen, H.H., and van de Kamp, J.J. (1987). Dicarboxylicaciduria and secondary carnitine deficiency in glycogenosis type IV. *Arch Dis Child* 62(10)**,** 1066-1067. doi: 10.1136/adc.62.10.1066.

Magoulas, P.L., El-Hattab, A.W., Roy, A., Bali, D.S., Finegold, M.J., and Craigen, W.J. (2012). Diffuse reticuloendothelial system involvement in type IV glycogen storage disease with a novel GBE1 mutation: a case report and review. *Hum Pathol* 43(6)**,** 943-951. doi: 10.1016/j.humpath.2011.10.001.

Malfatti, E., Barnerias, C., Hedberg-Oldfors, C., Gitiaux, C., Benezit, A., Oldfors, A., et al. (2016). A novel neuromuscular form of glycogen storage disease type IV with arthrogryposis, spinal stiffness and rare polyglucosan bodies in muscle. *Neuromuscul Disord* 26(10)**,** 681-687. doi: 10.1016/j.nmd.2016.07.005.

Marangoni, M., Smits, G., Ceysens, G., Costa, E., Coulon, R., Daelemans, C., et al. (2022). Implementation of fetal clinical exome sequencing: Comparing prospective and retrospective cohorts. *Genet Med* 24(2)**,** 344-363. doi: 10.1016/j.gim.2021.09.016.

Marchesi, C., Pagliarani, S., Lucchiari, S., Morandi, L., Salsano, E., Savoiardo, M., et al. (Year). "Phenotypic heterogeneity of GBE1 mutations: congenital glycogen storage disease type IV and adult polyglucosan body disease", in: *Meeting of the European Neurological Society*).

Maruyama, K., Suzuki, T., Koizumi, T., Sugie, H., Fukuda, T., Ito, M., et al. (2004). Congenital form of glycogen storage disease type IV: a case report and a review of the literature. *Pediatr Int* 46(4)**,** 474-477. doi: 10.1111/j.1442-200x.2004.01916.x.

Massey, C.N., and Dannaway, D. (2019). Glycogen storage disease type IV: Clinical presentation and diagnosis in a neonate. *Journal of Investigative Medicine* 67(2)**,** 524. doi: 10.1136/jim-2018-000974.439.

Mastrocola, F., Oliveira, W.S.d., Porto, A.A., Mendonça, R.M., and Oliveira Neto, N.R.d. (2020). Non-Progressive Hepatic Form of Andersen Disease as a Mimic of Hypertrophic Cardiomyopathy. *Arquivos Brasileiros de Cardiologia* 115**,** 964-966.

Matern, D., Starzl, T.E., Arnaout, W., Barnard, J., Bynon, J.S., Dhawan, A., et al. (1999). Liver transplantation for glycogen storage disease types I, III, and IV. *Eur J Pediatr* 158 Suppl 2**,** S43-48. doi: 10.1007/pl00014320.

McConkie-Rosell, A., Wilson, C., Piccoli, D.A., Boyle, J., DeClue, T., Kishnani, P., et al. (1996). Clinical and laboratory findings in four patients with the non-progressive hepatic form of type IV glycogen storage disease. *J Inherit Metab Dis* 19(1)**,** 51-58. doi: 10.1007/BF01799348.

McMaster, K., Powers, J., Hennigar Jr, G., Wohltmann, H., and Farr Jr, G. (1979). Nervous system involvement in type IV glycogenosis. *Archives of pathology & laboratory medicine* 103(3)**,** 105-111.

Michelakakis, H., Dimitriou, E., and Labadaridis, I. (2004). The expanding spectrum of disorders with elevated plasma chitotriosidase activity: an update. *J Inherit Metab Dis* 27(5)**,** 705-706. doi: 10.1023/b:boli.0000043025.17721.fc.

Mizuochi, T., Kimura, A., Nishiura, H., Inomata, Y., Okajima, H., Sugie, H., et al. (2011). Liver biopsy is an important procedure in the diagnosis of glycogen storage disease type IV. *Pediatr Int* 53(1)**,** 129-130. doi: 10.1111/j.1442-200X.2010.03282.x.

Morioka, D., Kasahara, M., Takada, Y., Corrales, J.P., Yoshizawa, A., Sakamoto, S., et al. (2005). Living donor liver transplantation for pediatric patients with inheritable metabolic disorders. *Am J Transplant* 5(11)**,** 2754-2763. doi: 10.1111/j.1600-6143.2005.01084.x.

Mortimer, P.E. (1965). Glycogen storage disease. *Proc R Soc Med* 58(9)**,** 700-701. doi: 10.1177/003591576505800918.

Nambu, M., Kawabe, K., Fukuda, T., Okuno, T.B., Ohta, S., Nonaka, I., et al. (2003). A neonatal form of glycogen storage disease type IV. *Neurology* 61(3)**,** 392-394.

Nase, S., Kunze, K.P., Sigmund, M., Schroeder, J.M., Shin, Y., and Hanrath, P. (1995). A new variant of type IV glycogenosis with primary cardiac manifestation and complete branching enzyme deficiency. In vivo detection by heart muscle biopsy. *Eur Heart J* 16(11)**,** 1698-1704. doi: 10.1093/oxfordjournals.eurheartj.a060797.

Ndugga-Kabuye, M.K., Maleszewski, J., Chanprasert, S., and Smith, K.D. (2019). Glycogen storage disease type IV: dilated cardiomyopathy as the isolated initial presentation in an adult patient. *BMJ Case Rep* 12(9). doi: 10.1136/bcr-2019-230068.

Nolte, K.W., Janecke, A.R., Vorgerd, M., Weis, J., and Schroder, J.M. (2008). Congenital type IV glycogenosis: the spectrum of pleomorphic polyglucosan bodies in muscle, nerve, and spinal cord with two novel mutations in the GBE1 gene. *Acta Neuropathol* 116(5)**,** 491-506. doi: 10.1007/s00401-008-0417-8.

Onal, I.K., Turhan, N., Oztas, E., Arhan, M., Akcoren, Z., Oguz, P., et al. (2009). Hepatocellular carcinoma in an adult patient with type IV glycogen storage disease. *Acta Gastroenterol Belg* 72(3)**,** 377-378.

Pan, S., Zu, S., and Yue, M. (2018). Clinical characteristics and gene mutation analysis of one pedigree with glycogen storage disease type Ⅳ. *Biomedical Research* 29(10)**,** 2160-2163.

Penchansky, L., Agostini, R., and Jaffe, R. (1992). Leukocyte inclusions in glycogen storage disease, type IV. *Pediatric pathology* 12(6)**,** 903-905.

Radhakrishnan, P., Moirangthem, A., Nayak, S.S., Shukla, A., Mathew, M., and Girisha, K.M. (2019). Novel pathogenic variants in GBE1 causing fetal akinesia deformation sequence and severe neuromuscular form of glycogen storage disease type IV. *Clin Dysmorphol* 28(1)**,** 17-21. doi: 10.1097/MCD.0000000000000248.

Raju, G.P., Li, H.C., Bali, D.S., Chen, Y.T., Urion, D.K., Lidov, H.G., et al. (2008). A case of congenital glycogen storage disease type IV with a novel GBE1 mutation. *J Child Neurol* 23(3)**,** 349-352. doi: 10.1177/0883073807309248.

Ravenscroft, G., Thompson, E.M., Todd, E.J., Yau, K.S., Kresoje, N., Sivadorai, P., et al. (2013). Whole exome sequencing in foetal akinesia expands the genotype-phenotype spectrum of GBE1 glycogen storage disease mutations. *Neuromuscul Disord* 23(2)**,** 165-169. doi: 10.1016/j.nmd.2012.11.005.

Reed, G.B., Jr., Dixon, J.F., Neustein, J.B., Donnell, G.N., and Landing, B.H. (1968). Type IV glycogenosis. Patient with absence of a branching enzyme alpha-1,4-glucan:alpha-1,4-glucan 6-glycosyl transferase. *Lab Invest* 19(5)**,** 546-557.

Reusche, E., Aksu, F., Goebel, H.H., Shin, Y.S., Yokota, T., and Reichmann, H. (1992). A mild juvenile variant of type IV glycogenosis. *Brain Dev* 14(1)**,** 36-43. doi: 10.1016/s0387-7604(12)80277-4.

Romano, F., Stroppa, P., Bravi, M., Casotti, V., Lucianetti, A., Guizzetti, M., et al. (2011). Favorable outcome of primary liver transplantation in children with cirrhosis and hepatocellular carcinoma. *Pediatr Transplant* 15(6)**,** 573-579. doi: 10.1111/j.1399-3046.2011.01528.x.

Rosenthal, P., Podesta, L., Grier, R., Said, J.W., Sher, L., Cocjin, J., et al. (1995). Failure of liver transplantation to diminish cardiac deposits of amylopectin and leukocyte inclusions in type IV glycogen storage disease. *Liver Transpl Surg* 1(6)**,** 373-376. doi: 10.1002/lt.500010607.

Sahoo, S., Blumberg, A.K., Sengupta, E., and Hart, J. (2002). Type IV glycogen storage disease. *Arch Pathol Lab Med* 126(5)**,** 630-631. doi: 10.5858/2002-126-0630-TIGSD.

Said, S.M., Murphree, M.I., Mounajjed, T., El-Youssef, M., and Zhang, L. (2016). A novel GBE1 gene variant in a child with glycogen storage disease type IV. *Hum Pathol* 54**,** 152-156. doi: 10.1016/j.humpath.2016.03.021.

Sandhu, T., Polan, M., Yu, Z., Lu, R., and Makkar, A. (2019). Case of Neonatal Fatality from Neuromuscular Variant of Glycogen Storage Disease Type IV. *JIMD Rep* 45**,** 51-55. doi: 10.1007/8904_2018_142.

Santonja, C., Moreno, A., Suarez, L., and Escobar, H. (1990). Liver biopsy diagnosis of Andersen's disease. *J Pediatr Gastroenterol Nutr* 10(2)**,** 274. doi: 10.1097/00005176-199002000-00028.

Santra, S., Chakrapani, A., Vijay, S., Mckiernan, P., Van Mourik, I., Kelly, D., et al. (2013). Fulminant hepatic failure in an infant with glycogen storage disease type IV. *Journal of Inherited Metabolic Disease* 36(Suppl 2)**,** S226.

Sarkar, A.K., Sarkar, S., Asawa, V., and Munshi, A.K. (1994). Glycogen storage disease (type-IV) with cirrhosis and metastatic intrathoracic neuroblastoma. *Indian J Pediatr* 61(3)**,** 293-297. doi: 10.1007/BF02752228.

Schanzer, A., Faas, D., Rust, S., Podskarbi, T., van Kuilenburg, A.B., Scarpa, M., et al. (2016). Distinctly Elevated Chitotriosidase Activity in a Child with Congenital Andersen Disease (Glycogen Storage Disease Type IV). *Klin Padiatr* 228(5)**,** 277-279. doi: 10.1055/s-0042-109399.

Schene, I.F., Korenke, C.G., Huidekoper, H.H., van der Pol, L., Dooijes, D., Breur, J., et al. (2019). Glycogen Storage Disease Type IV: A Rare Cause for Neuromuscular Disorders or Often Missed? *JIMD Rep* 45**,** 99-104. doi: 10.1007/8904_2018_148.

Schochet Jr, S., McCormick, W., and Zellweger, H. (1970). Type IV glycogenosis (amylopectinosis). Light and electron microscopic observations. *Archives of Pathology* 90(4)**,** 354-363.

Schroder, J.M., May, R., Shin, Y.S., Sigmund, M., and Nase-Huppmeier, S. (1993). Juvenile hereditary polyglucosan body disease with complete branching enzyme deficiency (type IV glycogenosis). *Acta Neuropathol* 85(4)**,** 419-430. doi: 10.1007/BF00334454.

Selby, R., Starzl, T.E., Yunis, E., Brown, B.I., Kendall, R.S., and Tzakis, A. (1991). Liver transplantation for type IV glycogen storage disease. *N Engl J Med* 324(1)**,** 39-42. doi: 10.1056/nejm199101033240107.

Selby, R., Starzl, T.E., Yunis, E., Todo, S., Tzakis, A.G., Brown, B.I., et al. (1993). Liver transplantation for type I and type IV glycogen storage disease. *Eur J Pediatr* 152 Suppl 1**,** S71-76. doi: 10.1007/BF02072093.

Servidei, S., Riepe, R.E., Langston, C., Tani, L.Y., Bricker, J.T., Crisp-Lindgren, N., et al. (1987). Severe cardiopathy in branching enzyme deficiency. *J Pediatr* 111(1)**,** 51-56. doi: 10.1016/s0022-3476(87)80341-4.

Shen, J., Liu, H.M., McConkie-Rosell, A., and Chen, Y.T. (1999). Prenatal diagnosis of glycogen storage disease type IV using PCR-based DNA mutation analysis. *Prenat Diagn* 19(9)**,** 837-839. doi: 10.1002/(sici)1097-0223(199909)19:9<837::aid-pd652>3.0.co;2-g.

Sidbury Jr, J., Mason, J., Burns Jr, W., and Ruebner, B. (1962). Type IV glycogenosis. Report of a case proven by characterization of glycogen and studied at necropsy. *Bulletin of the Johns Hopkins Hospital* 111**,** 157-181.

Sluiter, W., van den Bosch, J.C., Goudriaan, D.A., van Gelder, C.M., de Vries, J.M., Huijmans, J.G., et al. (2012). Rapid ultraperformance liquid chromatography-tandem mass spectrometry assay for a characteristic glycogen-derived tetrasaccharide in Pompe disease and other glycogen storage diseases. *Clin Chem* 58(7)**,** 1139-1147. doi: 10.1373/clinchem.2011.178319.

Sokal, E.M., Van Hoof, F., Alberti, D., de Ville de Goyet, J., de Barsy, T., and Otte, J.B. (1992). Progressive cardiac failure following orthotopic liver transplantation for type IV glycogenosis. *Eur J Pediatr* 151(3)**,** 200-203. doi: 10.1007/bf01954384.

Soto, J., Giraldo, L., and Sanchez, A. (2017). Exome sequencing for the clinical diagnosis of non-progressive hepatic form of glycogen storage disease IV; Abstracts presented at the 13th International Congress of Inborn Errors of Metabolism - ICIEM 2017. *Journal of Inborn Errors of Metabolism and Screening* 5(370).

Sreekantam, S., Rizvi, H., Brown, R., Santra, S., Raiman, J., Vijay, S., et al. (2020). An uncommon cause of early infantile liver disease and raised chitotriosidase. *JIMD Rep* 54(1)**,** 22-24. doi: 10.1002/jmd2.12123.

Starzl, T.E., Demetris, A.J., Trucco, M., Ricordi, C., Ildstad, S., Terasaki, P.I., et al. (1993). Chimerism after liver transplantation for type IV glycogen storage disease and type 1 Gaucher's disease. *N Engl J Med* 328(11)**,** 745-749. doi: 10.1056/NEJM199303183281101.

Stonko, D.P., and Crook, T.W. (2017). A 10-Month-Old Male With a Cough, Fever, and Abnormal Hepatitis Serologies. *Clin Pediatr (Phila)* 56(6)**,** 593-595. doi: 10.1177/0009922816656630.

Szymanska, E., Szymanska, S., Truszkowska, G., Ciara, E., Pronicki, M., Shin, Y.S., et al. (2018). Variable clinical presentation of glycogen storage disease type IV: from severe hepatosplenomegaly to cardiac insufficiency. Some discrepancies in genetic and biochemical abnormalities. *Arch Med Sci* 14(1)**,** 237-247. doi: 10.5114/aoms.2018.72246.

Tang, T.T., Segura, A.D., Chen, Y.T., Ricci, L.M., Franciosi, R.A., Splaingard, M.L., et al. (1994). Neonatal hypotonia and cardiomyopathy secondary to type IV glycogenosis. *Acta Neuropathol* 87(5)**,** 531-536. doi: 10.1007/BF00294181.

Taratuto, A.L., Akman, H.O., Saccoliti, M., Riudavets, M., Arakaki, N., Mesa, L., et al. (2010). Branching enzyme deficiency/glycogenosis storage disease type IV presenting as a severe congenital hypotonia: muscle biopsy and autopsy findings, biochemical and molecular genetic studies. *Neuromuscul Disord* 20(12)**,** 783-790. doi: 10.1016/j.nmd.2010.07.275.

Tay, S.K., Akman, H.O., Chung, W.K., Pike, M.G., Muntoni, F., Hays, A.P., et al. (2004). Fatal infantile neuromuscular presentation of glycogen storage disease type IV. *Neuromuscul Disord* 14(4)**,** 253-260. doi: 10.1016/j.nmd.2003.12.006.

Tunon, T., Bengoechea, O., and Narbona, J. (1988). Glycogenosis with amylopectinoid deposits in a 13-year-old girl. *Clin Neuropathol* 7(3)**,** 100-104.

van Noort, G., Straks, W., Van Diggelen, O.P., and Hennekam, R.C. (1993). A congenital variant of glycogenosis type IV. *Pediatr Pathol* 13(5)**,** 685-698. doi: 10.3109/15513819309048254.

Walter, M.C., Wenninger, S., and Abicht, A. (2018). Glycogen storage disease type iv presenting as congenital myopathy with contractures and rigid spine. *Journal of Neuromuscular Diseases* 5 Supplement 1.

Wei, A., Ma, H., Li, Z., Zhang, L., Zhang, R., and Wang, T. (2020). Type IV Glycogen Storage Disease Associated With Hemophagocytic Lymphohistiocytosis: A Case Report. *J Pediatr Hematol Oncol* 42(5)**,** 368-369. doi: 10.1097/MPH.0000000000001694.

Willot, S., Marchand, V., Rasquin, A., Alvarez, F., and Martin, S.R. (2010). Systemic progression of type IV glycogen storage disease after liver transplantation. *J Pediatr Gastroenterol Nutr* 51(5)**,** 661-664. doi: 10.1097/MPG.0b013e3181d29780.

Ying, S., Zhihua, Z., Yucan, Z., Yu, J., Qian, L., Bixia, Z., et al. (2020). Molecular Diagnosis of Panel-Based Next-Generation Sequencing Approach and Clinical Symptoms in Patients With Glycogen Storage Disease: A Single Center Retrospective Study. *Front Pediatr* 8**,** 600446. doi: 10.3389/fped.2020.600446.

Yu, W., Brundler, M.A., and Wright, J.R., Jr. (2018). Polyglucosan Bodies in Placental Extravillious Trophoblast for the Diagnosis of Fatal Perinatal Neuromuscular-type Glycogen Storage Disease Type IV. *Pediatr Dev Pathol* 21(4)**,** 423-427. doi: 10.1177/1093526617707852.

Zellweger, H., Mueller, S., Ionasescu, V., Schochet, S.S., and McCormick, W.F. (1972). Glycogenosis. IV. A new cause of infantile hypotonia. *J Pediatr* 80(5)**,** 842-844. doi: 10.1016/s0022-3476(72)80144-6.
