# Supplementary material for: A novel approach to characterize phenotypic variation in GSD IV: Reconceptualizing the clinical continuum
Source: Front Genet. 2022 Sep 13;13:992406. doi: 10.3389/fgene.2022.992406 (PMC9513518; doi:10.3389/fgene.2022.992406)
Supplement: Supplementary file 2 [file DataSheet1.DOCX]

**Appendix 1. GSD IV Systematic Review Search Strategy.** Designed by Samantha Kaplan, PhD.

**Database: MEDLINE via Ovid (371 results)**

exp "Glycogen Storage Disease Type IV"/ OR (("Glycogen Storage Disease".tw. OR GSD.tw.) adj2 (IV.tw. OR four.tw.)) OR GSDIV.tw. OR GSD4.tw. OR ((glycogenosis.tw. OR glycogenoses.tw.) adj2 (4.tw. OR IV.tw.)) OR "Andersen disease".tw. OR "Andersens disease".tw. OR "Andersen s disease".tw. OR "Andersen's disease".tw. OR "brancher deficiency".tw. OR "brancher deficiencies".tw. OR "Gbe 1 deficiency".tw. OR "Gbe 1 deficiencies".tw. OR Amylopectinoses.tw. OR Amylopectinosis.tw. OR APBD.tw. OR ((adult.tw.) adj2 (polyglucosan.tw.) adj2 (body.tw.) adj2 (disease.tw.))

**Database: Embase via Elsevier (656 results)**

'glycogen storage disease type 4'/exp OR (("Glycogen Storage Disease" OR GSD) NEAR/2 (IV OR four)) OR GSDIV: OR GSD4:ti,ab OR ((glycogenosis OR glycogenoses) NEAR/2 (4 OR IV)) OR "Andersen disease":ti,ab OR "Andersens disease":ti,ab OR "Andersen s disease":ti,ab OR "brancher deficiency":ti,ab OR "brancher deficiencies":ti,ab OR "Gbe 1 deficiency":ti,ab OR "Gbe 1 deficiencies":ti,ab OR Amylopectinoses:ti,ab OR Amylopectinosis:ti,ab OR APBD:ti,ab OR ((adult) NEAR/2 (polyglucosan) NEAR/2 (body) NEAR/2 (disease))

**Database: Scopus via Elsevier (477 results)**

TITLE-ABS-KEY( (("Glycogen Storage Disease" OR GSD) W/2 (IV OR four)) OR GSDIV OR GSD4 OR ((glycogenosis OR glycogenoses) W/2 (4 OR IV)) OR "Andersen disease" OR "Andersens disease" OR "Andersen s disease" OR "Andersen's disease" OR "brancher deficiency" OR "brancher deficiencies" OR "Gbe 1 deficiency" OR "Gbe 1 deficiencies" OR Amylopectinoses OR Amylopectinosis OR APBD OR ((adult) W/2 (polyglucosan) NEAR/2 (body) W/2 (disease)))
